# Diffusion-based representation integration for foundation models improves spatial transcriptomics analysis

**DOI:** 10.1093/bioinformatics/btag259

**Published:** 2026-07-07

**Authors:** Atishay Jain, Tuan M Pham, David H Laidlaw, Ying Ma, Ritambhara Singh

**Affiliations:** Department of Computer Science, Brown University, Providence, RI 02912, United States; Center for Computational Molecular Biology, Brown University, Providence, RI 02912, United States; Department of Computer Science, Brown University, Providence, RI 02912, United States; Center for Computational Molecular Biology, Brown University, Providence, RI 02912, United States; Department of Biostatistics, Brown University, Providence, RI 02903, United States; Department of Computer Science, Brown University, Providence, RI 02912, United States; Center for Computational Molecular Biology, Brown University, Providence, RI 02912, United States

## Abstract

**Motivation:**

We propose DRIFT, a framework that integrates spatial context into the input representations for foundation models by leveraging diffusion on spatial graphs derived from spatial transcriptomics (ST) data. ST captures gene expression profiles while preserving spatial context, enabling downstream analysis tasks such as cell-type annotation, clustering, and cross-sample alignment. However, due to its emerging nature, there are very few foundation models that can utilize ST data to generate embeddings generalizable across multiple tasks. Meanwhile, well-documented foundational models trained on large-scale single-cell gene expression (scRNA-seq) data have demonstrated generalizable performance across scRNA-seq assays, tissues, and tasks; however, they do not leverage the spatial information in ST data. We use heat kernel diffusion to propagate embeddings across spatial neighborhoods, incorporating the local neighborhood context of the ST data while preserving the transcriptomic representations learned by state-of-the-art single-cell foundation models.

**Results:**

We systematically benchmark five foundational models (both scRNA-seq and ST-based) across key ST tasks such as annotation, alignment, and clustering, ensuring a comprehensive evaluation of our proposed framework. Our results show that DRIFT significantly improves the performance of existing foundational models on ST data over specialized state-of-the-art methods. Overall, DRIFT is an effective, accessible, and generalizable framework that bridges the gap toward universal models for modeling spatial transcriptomics.

**Availability and implementation:**

Code and data are available at https://github.com/rsinghlab/DRIFT.

## 1 Introduction

Spatial transcriptomics (ST) technologies enable measurement of gene expression while preserving tissue architecture, offering a direct view of how cellular identity and spatial organization together shape biological function. By jointly capturing transcriptomic and spatial information, ST datasets reveal the local microenvironment of gene activity and support downstream analyses such as spatially variable gene detection (Kats *et al.* 2021, Zhu *et al.* 2021, Adhikari *et al.* 2024, Chen *et al.* 2024a, Yuan *et al.* 2024a), clustering (Dong and Zhang 2022, Long *et al.* 2023, Yuan *et al.* 2024b, Zhang *et al.* 2024), cell–cell interaction inference (Garcia-Alonso *et al.* 2021, Cang *et al.* 2023, Yang *et al.* 2024, Jin *et al.* 2025), and cross-section alignment (Zeira *et al.* 2022, Liu *et al.* 2023). These capabilities have made ST an essential approach in developmental biology, pathology, and tissue-level systems analysis. However, the rapidly growing complexity and heterogeneity of ST data necessitate generalizing and automating the downstream tasks across a wide range of tissues, species, and technologies. Furthermore, ST measurements are typically sparse, noisy, and less throughput-efficient than single-cell RNA sequencing (scRNA-seq). This highlights the need for robust computational models that can extract biologically meaningful and spatially aware representations that generalize to diverse downstream ST analyses.

Recently, to address these challenges, ST foundation models have emerged, marking a growing effort to bring foundation modeling into the ST domain. Early examples include Loki (Chen *et al.* 2025), Nicheformer (Schaar *et al.* 2024), SToFM (Zhao *et al.* 2025), and STFormer (Li *et al.* 2025). Loki aligns histology images and spatial gene-expression profiles through cross-modal contrastive learning; Nicheformer applies a transformer trained on ST data, where attention mechanisms capture neighborhood-level dependencies, and SToFM and STFormer introduce spatial attention and positional encoding schemes to jointly model biological and spatial information. However, they rely heavily on large paired multimodal datasets (Chen *et al.* 2025) or cell-type priors derived from deconvolution (Li *et al.* 2025), which are often unavailable for many ST platforms. Moreover, some models do not explicitly model spatial relationships, limiting their ability to capture cell neighborhoods (Schaar *et al.* 2024, Chen *et al.* 2025). Additionally, training these architectures from scratch is computationally expensive, often requiring substantial computing resources. Finally, existing ST foundation models are trained on relatively homogeneous and small ST datasets. As a result, these models typically require dataset-specific retraining, limiting their utility as universal foundation models for ST datasets.

On the other hand, scRNA-seq foundation models are trained on millions of scRNA-seq profiles from diverse tissues and species. Models such as Geneformer (Theodoris *et al.* 2023, Chen *et al.* 2024b), scGPT (Cui *et al.* 2024), and scFoundation (Hao *et al.* 2024) learn gene–gene dependencies using transformer-based or masked language-model architectures. The embeddings generated by these models capture regulatory patterns, providing general-purpose representations that can transfer effectively across datasets and biological contexts. Their success demonstrates that large pretrained models can generalize across data sources and experimental conditions with limited fine-tuning, offering a promising foundation for diverse multi-tissue and cross-species analyses (Wu *et al.* 2025). However, since these models are trained solely on scRNA-seq data, they lack the spatial context critical for modeling ST data.

We introduce **D**iffusion-based **R**epresentation **I**ntegration for **F**oundation models in spatial **T**ranscriptomics (**DRIFT**). DRIFT constructs a spatial adjacency graph among tissue spots (or cells) and applies a heat-kernel diffusion process that propagates gene-expression signals across local neighborhoods. These smoothing produces spatially coherent and denoised gene expression representations that can be directly fed into any pretrained foundation model without retraining, making our approach much more computationally scalable and accessible. Foundation models that do not explicitly model neighborhood information benefit from both spatial context integration and denoising, while methods that do so leverage DRIFT’s denoised input.

We demonstrate that DRIFT generalizes easily across three critical ST tasks: cell-type annotation, cross-section alignment, and clustering. A single DRIFT framework outperforms state-of-the-art methods specialized for the different ST tasks, thereby setting a new norm for ST downstream analysis. Furthermore, the DRIFT diffusion leverages existing pretrained scRNA-seq foundation models, achieving improved performance compared to baseline ST foundation models. Therefore, our results establish that the diffusion operator at the core of DRIFT provides a simple, efficient, and general way to couple pretrained scRNA-seq foundation models with ST data by incorporating a spatially aware representation. For ST foundation models that may embed spatial context, diffusion-based denoising still improves performance. DRIFT’s generalizability across technologies, tissues, and models underscores its robustness for the evolving ST field.

## 2 Materials and methods

**Figure 1:**
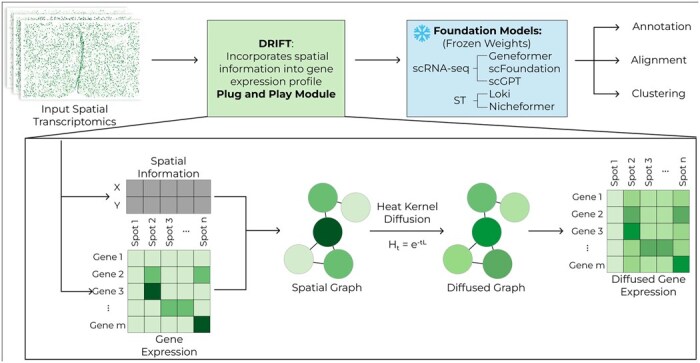
An illustration of the DRIFT Framework. DRIFT is a plug-and-play module that is applied to frozen foundation models and requires much less computational resources in comparison to training them. Gene expression profiles of individual spots lack spatial context. Therefore, we create a spatial graph in which each node represents a cell/spot, and each edge represents the spatial proximity between the corresponding nodes. We then apply a mathematically principled heat-kernel graph diffusion on the constructed graph and use the resulting diffused gene expression profiles for each cell/spot. Through diffusion, we perform a soft low-pass filtering that propagates spatial context and denoises the data. The diffused gene expression values are fed into foundation models instead of the original gene expression for downstream tasks.

As illustrated in Fig. 1, we introduce a graph diffusion-based framework, DRIFT, to incorporate neighborhood information into the gene expression profiles. To perform this integration, we construct a spatial graph and apply heat kernel diffusion over it. Our method operates on the premise that spatially proximal cells/spots likely share cell-type identity, allowing us to denoise the data by propagating signal across local neighborhoods (Pierson *et al.* 2015, Sonawane *et al.* 2017, Yu *et al.* 2025). The diffused gene expression matrices obtained from DRIFT are used as inputs to pretrained foundation models, which are then evaluated for multiple important ST analysis tasks over a variety of ST datasets.

### 2.1 DRIFT approach

First, we represent the ST data as a graph G=(V,E), where each node $v_{i}\in V$ corresponds to a single tissue location in the ST dataset (i.e. a cell or spot). Edges $(v_{i},v_{j})\in E$ connect spatially proximal nodes based on Euclidean distance. This graph construction strategy is applied uniformly across both cell-resolution and spot-resolution datasets. Following an empirical evaluation of construction strategies (detailed in Section 1, available as supplementary data at *Bioinformatics* online), we implemented a *k*-nearest neighbor graph with k=3, which yielded the most robust performance across datasets irrespective of ST platform resolution. The edges of this graph are represented by the adjacency matrix $A\in R^{n\times n}$, where $A_{ij}=1$ if nodes *i* and *j* are spatial neighbors and $A_{ij}=0$ otherwise.

We then calculate the degree matrix *D*, which is a diagonal matrix defined as


(1) $$D_{ii}=\sum_{j}A_{ij},$$

representing the degree of each node *i*. Using these, we compute the graph Laplacian as


(2) L=D−A.

The Laplacian encodes the structure of *G* and serves as a discrete analogue of the continuous Laplace operator, capturing how information flows between connected nodes.

Heat kernel diffusion models the propagation of information (or “heat”) across a graph. It is defined by the heat equation on graphs:


(3) $$\frac{dx_{t}}{dt}=-Lx_{t},$$

Solving this differential equation yields the heat kernel solution:


(4) $$x(t)=e^{-tL}x_{0},$$

where, $x_{0}$ represents the original (non-diffused) gene expression matrix and $x_{t}$ denotes the diffused gene expression matrix at time *t*. *t* is therefore a hyperparameter that controls the strength of diffusion. Small *t* values (representing a shorter time for heat diffusion) lead to weaker diffusion and preserve local structure. Meanwhile, large *t* values result in stronger smoothing across larger spatial distances.

#### 2.1.1 Relation to spectral graph theory

Since *G* is an undirected graph, its Laplacian *L* is a symmetric real matrix and is therefore diagonalizable. By the graph spectral theorem, it can be decomposed as


(5) $$L=U\Lambda U^{-1}=U\Lambda U^{⊤},$$

where, *U* is the orthogonal matrix of eigenvectors and Λ is the diagonal matrix of the corresponding eigenvalues. Substituting Equation (5) into the heat kernel [Equation (4)] gives:


(6) $$e^{-tL}=Ue^{-t\Lambda }U^{⊤}.$$

This expression reveals that diffusion corresponds to exponentially decaying the eigenvalues of the Laplacian, which is equivalent to a soft low-pass filter on the graph spectrum. Effectively, it smoothens gene expression based on the graph structure (Zhang and Hancock 2008), incorporating the cell’s neighborhood information. This idea has been previously explored for scRNA-seq (Van Dijk *et al.* 2018) and 3D genome organization data (Ursu *et al.* 2018, He *et al.* 2023), further demonstrating that graph diffusion techniques can effectively denoise data, smooth signals across neighboring nodes, and capture spatial relationships. The resulting diffused gene expression matrix x(t) thus integrates the spatial context of cells directly with the gene expression matrix, making it compatible with existing scRNA-seq foundation models that only accept gene expression values as inputs. Therefore, our choice of heat kernel diffusion is appropriate for our ST data modeling task. To further justify our choice of the heat kernel for graph diffusion, we show that it outperforms other diffusion and denoising methods overall (Section 2, available as supplementary data at *Bioinformatics* online).

Section 3, available as supplementary data at *Bioinformatics* online, validates the importance of spatial context via graph shuffling and neighborhood prediction benchmarks, confirming that DRIFT successfully incorporates this context into the input gene expression profiles. Next, we input the diffused gene expression matrix into the foundation models.

### 2.2 Foundation models

We apply DRIFT to a variety of scRNA-seq and ST foundation models to illustrate its ability to leverage the performance of existing models. We first demonstrate that DRIFT incorporates neighborhood information by pairing it with scRNA-seq foundation models. We further show its denoising property by enhancing the performance of ST foundation models.

#### 2.2.1 Geneformer

Geneformer is a scRNA-seq foundation model that has three models. V1 is trained on 30 million cells (Theodoris *et al.* 2023), while V2 is trained on 104 million cells (Chen *et al.* 2024b). Furthermore, V2 has two model architecture configurations: one with 104 million parameters and another with 316 million parameters. In our Section 4, available as supplementary data at *Bioinformatics* online, we demonstrate that V2, with 316 million parameters, consistently outperforms other Geneformer configurations. Therefore, for any further experiments, we select V2 with 316 million parameters as the Geneformer foundation model.

#### 2.2.2 scFoundation

scFoundation is a large-scale foundation model pretrained over 50 million single-cell transcriptomic profiles with an underlying encoder–decoder architecture.

#### 2.2.3 scGPT

scGPT is a scRNA-seq foundation model trained on over 33 million cells, simultaneously learning cell and gene embeddings. It utilizes a specially designed attention mask and generative training to jointly optimize both embeddings.

#### 2.2.4 Loki

Loki is a visual-omics foundation model that learns joint representations between histology images and transcriptomic profiles using contrastive learning based on OpenClip (Ilharco *et al.* 2021). It is trained on 2.2 million paired image-transcriptomic patches. The framework does not explicitly train spatial information.

#### 2.2.5 Nicheformer

Nicheformer is a transformer-based foundation model trained on 110 million single-cells and ST spots. By training on both scRNA-seq and ST, Nicheformer learns the characteristics of different dataset types. Nicheformer also does not explicitly use spatial information during training.

We provide additional technical context in Section 5, available as supplementary data at *Bioinformatics* online, including a brief explanation of how the foundation models handle varying gene sets across different ST platforms. The embeddings from these foundation models are then used in three important downstream ST tasks, as described below.

### 2.3 Spatial transcriptomic tasks and specialized methods

To demonstrate the advantage of DRIFT, we measure its performance improvement over baseline foundation models across multiple ST tasks. These tasks are crucial for analyzing ST data and also facilitate cell-atlas construction, understanding disease mechanisms, and biomedical discovery (Williams *et al.* 2022, Tian *et al.* 2023). We also compare the DRIFT-incorporated foundation models to well-documented state-of-the-art specialized methods for each task. We note that the selected specialized methods may rely on different assumptions or be tuned for specific data modalities or objectives. We include comparisons with these specialized methods primarily to demonstrate the versatile utility of DRIFT across a wide range of ST-related tasks.

#### 2.3.1 Cell-type annotation

The aim of cell-type annotation is to identify cell types in a supervised manner, given a reference dataset. This is critical for understanding the heterogeneity of tissues and related biological processes (Fan *et al.* 2023). Annotation is formulated as a few-shot learning task, where we attach a classification head (or probe) to a pretrained foundation model, and only train the weights of the probe to identify the cell types.

We compare against STELLAR (Brbić *et al.* 2022), a widely adopted deep-learning framework that has demonstrated superior annotation performance. STELLAR generates spatial graphs to represent ST data and uses a graph neural network (GNN) to learn embeddings of spots in an annotated reference ST dataset. Then it annotates cells in the unannotated ST dataset using the embeddings it generates. We assess the annotation performance through the accuracy and F1-score metrics.

#### 2.3.2 Slice alignment

ST slice alignment is essential for reconstructing coherent anatomical structures from adjacent ST sections, as these slices represent slightly different structures and are subject to technical rotation, stretching, or deformation. Evaluating alignment accuracy, therefore, provides a rigorous test of how well DRIFT-incorporated embeddings preserve spatial geometry and enable meaningful cross-slice correspondence. We treat this as a zero-shot setting, applying Coherent Point Drift (CPD) directly to the original and DRIFT-incorporated embeddings, without any task-specific fine-tuning, across three alignment contexts with varying levels of noise and distortion. Baseline task-specific methods are PASTE (Zeira *et al.* 2022) and PASTE2 (Liu *et al.* 2023). PASTE uses Fused Gromov–Wasserstein (FGW) alignment, returning probabilistic alignment, while PASTE2 extends this to partial FGW alignment to handle partial overlaps. They are best suited for spatial alignments for homogeneous datasets (i.e. across continuous slices) (Hu *et al.* 2024).

We evaluate alignment performance using DRIFT-incorporated embeddings across both simulated and real-world datasets. In the simulation experiment, we construct each cell/spot’s total count using a Negative Binomial fit to the real library-size mean and variance, then generate per-gene counts by drawing from a multinomial whose probabilities are the real gene proportions with a pseudocount δ added.

For simulated data, we evaluate alignment quality using translation error together with PCC and Kendall’s τ, since ground-truth spatial coordinates are available. For real-world datasets, where true spatial coordinates are unknown, we assess alignment through PCC, and Kendall’s τ.

*Translation Error—*The translation error, defined as $||t_{\mathrm{pred}}-t_{\mathrm{true}}|{|}_{2}$ measures the Euclidean discrepancy between the predicted translation vector and the ground-truth shift required for correct spatial alignment. A lower translation error indicates a more accurate alignment compared to the ground truth. Translation error is computed only when ground-truth spatial coordinates are available (simulated data).

*Pearson Correlation—*For each aligned pair, we compute the Pearson correlation coefficient (PCC) as follows:


$$\mathrm{PCC}=\frac{\sum_{i=1}^{G}\left(x_{i}-\bar{x}\right)(y_{i}-\bar{y})}{\sqrt{\sum_{i=1}^{G}{\left(x_{i}-\bar{x}\right)}^{2}}\sqrt{\sum_{i=1}^{G}{\left(y_{i}-\bar{y}\right)}^{2}}}.$$

PCC captures the global expression pattern and linear correspondence between aligned slices. We report the median PCC across all aligned pairs, with higher values indicating stronger transcriptomic consistency between matched cells.

*Kendall’s* τ - We quantify rank-based concordance between aligned cells using Kendall’s τ: $\tau =\frac{2(C-D)}{G(G-1)}$, where *C* and *D* are counts of concordant and discordant gene pairs, and a higher τ indicates stronger rank similarity. We report the median τ, with higher values suggesting biologically coherent correspondence between matched cells.

#### 2.3.3 Clustering

Unsupervised clustering is the task of grouping cells without any prior information, which is an essential step for identifying marker genes (Hu *et al.* 2021, Li *et al.* 2022), and understanding disease pathology (Chang *et al.* 2022, Chen *et al.* 2023). Since it does not explicitly require any labels for training, it is a zero-shot task in which embeddings obtained from the foundation models are directly fed into unsupervised clustering algorithms, such as mclust (Scrucca *et al.* 2016) (a finite normal mixture modeling-based clustering algorithm), to obtain the final clusters.

To benchmark clustering performance, we compared DRIFT against GraphST (Long *et al.* 2023), which has been identified as a top-performing method in recent independent evaluations (Hu *et al.* 2024, Yuan *et al.* 2024b). GraphST employs a GNN autoencoder architecture and contrastive learning techniques to learn embeddings that are subsequently utilized in unsupervised algorithms. We evaluate the methods using the adjusted Rand index (ARI). ARI is a metric commonly used to assess unsupervised clustering performance (Long *et al.* 2023, Hu *et al.* 2024, Yuan *et al.* 2024b) because it accounts for the risk of accidental cluster agreement.

### 2.4 Datasets

To demonstrate DRIFT’s generalizability, we benchmark it across eight datasets from various ST technologies and tissues of humans and mice. The ST technology, species, tissue, and number of slices for all the datasets are summarized in Table 1.

**Table 1:** A summary of all the datasets.^a^

Dataset	ST Platform	Species	Tissue	Number of Slices
10xHPC (Maynard *et al.* 2021)	10x Visium	Human	Prefrontal cortex	12
10xHOC (Villacampa *et al.* 2021)	10x Visium	Human	Ovarian cancer	2
10xHSI(Mirzazadeh *et al.* 2023)	10x Visium	Human	Small intestine	12
MERHH (Farah *et al.* 2024)	MERFISH	Human	Heart	3
MERMPH (Moffitt *et al.* 2018, Li and Zhou 2022)	MERFISH	Mouse	Preoptic hypothalamus	5
MERMB (Zhang *et al.* 2023)	MERFISH	Mouse	Brain	15
MERMBA (Allen *et al.* 2023)	MERFISH	Mouse	Aging brain	12
StereoME (Chen *et al.* 2022)	Stereo-Seq	Mouse	Embryo	4

a The table lists the ST technology, species, tissue, and number of slices in all the datasets.

### 2.5 Hyperparameter tuning

For every task, we tuned the *t* value (representing the strength of diffusion) in the heat kernel diffusion equation. The values assigned to *t* during hyperparameter tuning were 0.00001, 0.0001, 0.001, 0.01, 0.1, 1, 2, 5, 10, 100. We observed that *t* values from 1 to 10 performed similarly well overall. We suggest a default value of t=5 due to its high performance across tasks, datasets, and foundation models. Results for hyperparameter tuning *t* are summarized in Section 6, available as supplementary data at *Bioinformatics* online.

Any further hyperparameter tuning necessary for both foundation models and specialized methods during few-shot learning tasks was performed using Optuna (Akiba *et al.* 2019) with 10 trials. We also tuned PASTE and PASTE2 for the zero-shot alignment task. The hyperparameters and their value ranges are listed in Sections 7 and 8, available as supplementary data at *Bioinformatics* online.

## 3 Results

### 3.1 DRIFT demonstrates state-of-the-art cell-type annotation performance

We first tested DRIFT’s performance on the cell-type annotation task. We formulated it as a few-shot problem, with training performed on just one slice. For all foundation models, we attached a three-layer multilayer perceptron (MLP) classification head to predict cell-type labels, while freezing all their encoder weights. The MLP was trained using cross-entropy loss.

In this experiment, we employed datasets 10xHPC (10x human prefrontal cortex), MERHH (MERFISH human heart), MERMPH (MERFISH mouse preoptic hypothalamus), MERMB (MERFISH mouse brain), and MERMBA (MERFISH mouse brain) since they contain expert annotations necessary for evaluating the methods. For spot-resolution platforms, annotations represent the dominant cell type within the spot, as determined by the provided expert pathology annotations. The five foundation model classification heads and one specialized method (STELLAR) were trained and evaluated independently on each dataset. To recreate the realistic scenario of limited annotated data, we used only one annotated slice per dataset to train and hyperparameter-tune the methods, reserving the remaining slices for testing.

**Figure 2:**
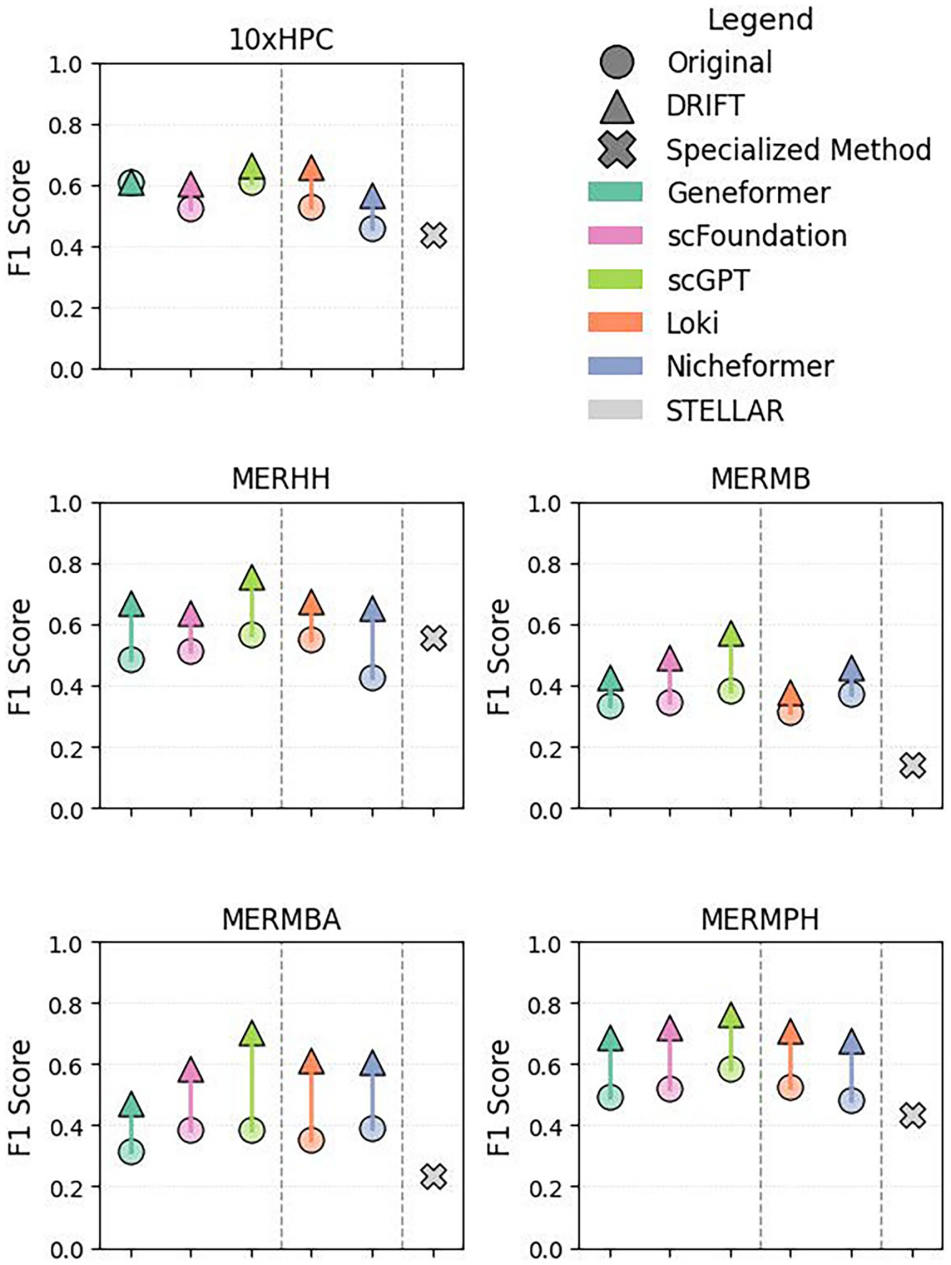
Cell-type annotation results. A quantitative comparison across multiple datasets. The circles represent the performance of the original foundation models, the triangles represent foundation models with DRIFT-enhanced inputs, and the X represents the specialized method STELLAR. The colors indicate which column corresponds to each foundation model (the order of models in the legend matches column placement). This figure shows that DRIFT improves the F1 score of foundation models on the annotation task. Furthermore, DRIFT-incorporated foundation models, specifically scGPT show state-of-the-art performance, surpassing even the specialized model—STELLAR.

Figure 2 displays DRIFT’s F1-score performance improvement across all methods and datasets, demonstrating that the heat kernel’s spatial context integration and denoising properties are beneficial for the annotation task. We first compared the original scRNA-seq foundation models to the corresponding DRIFT-incorporated foundation models. Compared to the original foundation models, DRIFT shows average improvements of 19.81%–32.5% in accuracy and 30.75%–40.57% in F1-score. Dataset-specific accuracy results also show the same performance trends and are plotted in Section 9, available as supplementary data at *Bioinformatics* online.

In particular, scGPT with DRIFT shows the highest performance over all datasets. Furthermore, it consistently outperforms the specialized method, STELLAR. scGPT with DRIFT shows, on average, a 73.32% improvement in accuracy, and a 131.89% improvement in F1-score. scGPT with DRIFT also outperforms the original ST-based Loki and Nicheformer models. When compared to Loki, DRIFT-incorporated scGPT shows an average accuracy improvement of 48.46% and an average F1-score improvement of 57.96%. Similarly, when compared to Nicheformer, it shows an average accuracy improvement of 50.86% and an average F1-score improvement of 62.65%. These results demonstrate its state-of-the-art performance for ST annotation.

DRIFT also shows an improved performance for both the ST foundation models. Compared with the original Loki model, DRIFT-incorporated Loki shows an average accuracy improvement of 32.01% and an average F1-score improvement of 35.28%. Similarly, for Nicheformer, DRIFT shows an average accuracy improvement of 30.67% and an average F1-score improvement of 38.88%. These results illustrate the effectiveness of DRIFT in enhancing the ST foundation models.

To further explore the effects of DRIFT on spatial data, we performed comparative spatial boundary and rare cell type analyses. Results improves global structures and enhances cell identification via marker gene expression, including for low-abundance cell types while largely preserving underlying spatial organization (Section 10, available as supplementary data at *Bioinformatics* online).

### 3.2 DRIFT improves ST alignment across different contexts

For alignment tasks, we set up three experiments on one simulated dataset and two real datasets, and applied the Coherent Point Drift (CPD) algorithm (Myronenko and Song 2010) to align embeddings from the five foundational models, with and without DRIFT. We also evaluated the results using two specialized methods, PASTE and PASTE2. We formulated all the tasks as zero-shot problems, with no re-training or fine-tuning. After alignment, we measure the similarity between the expression profiles of the paired cells using the metrics described in the Methods section.

#### 3.2.1 Simulations

**Figure 3:**
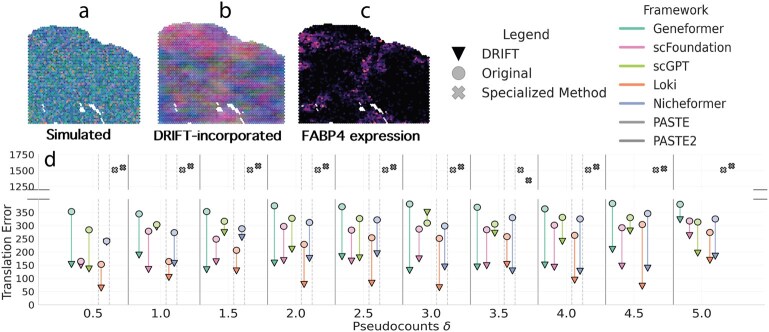
Simulated data alignment results. (a–c) PCA visualizations of the original and DRIFT-incorporated embeddings for simulated data at pseudocount 4.0, along with the corresponding FABP4 expression patterns. DRIFT produces embeddings with clearer and more coherent spatial organization, recovering biologically meaningful structure associated with ovarian cancer development. (d) Translation error across simulated datasets at different values of pseudocounts, demonstrating improved alignment fidelity after applying DRIFT.

To assess DRIFT’s robustness to noise, we simulated 10 paired ST slices from a manually aligned 10xHSI (Visium human ovarian cancer) pair, varying the pseudocount level δ (from 0.5 to 5) to introduce increased expression noise [as done in Liu *et al.* (2023), Hu *et al.* (2024)]. We compared DRIFT and baseline alignments using translation error (lower is better), Pearson Correlation, and Kendall’s τ (higher is better). Across all pseudocount settings, DRIFT consistently reduces translation error in most scenarios (Fig. 3). For scRNA-seq foundation models, Geneformer and scFoundation exhibit stable and substantial reduction, typically ranging from 15% to 66% and frequently exceeding 50%. scGPT shows more variable behavior, achieving moderate improvements (10%–52%) in most settings. Spatial foundation models demonstrate particularly strong benefits, with Loki achieving the largest improvements (up to 76.9%) and Nicheformer showing consistent reduction of 40%–61% across most pseudocount scenarios. This positive improvement is further supported by systematically higher PCC values and Kendall’s τ between 0.5% and 4% gains. All DRIFT-enhanced results outperformed PASTE and PASTE2, suggesting the versitile use of the diffused embeddings for alignment tasks with noisy data (Fig. 22, available as supplementary data at *Bioinformatics* online) (Fig. 3)

#### 3.2.2 Mouse embryonic dataset

**Figure 4:**
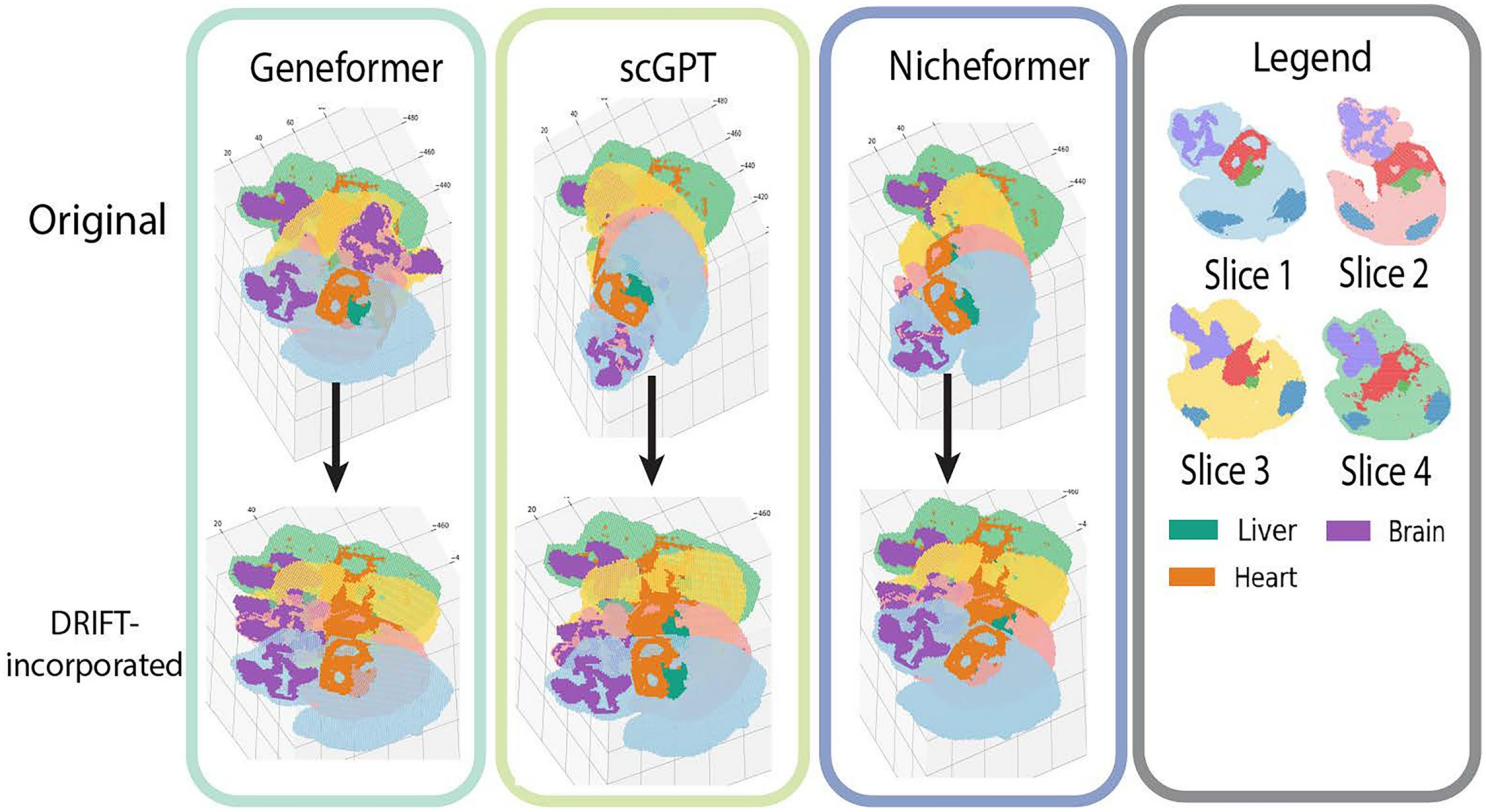
Select mouse embryo alignment results. First row: alignments obtained using the original embeddings of Geneformer, scGPT, and Nichefomer. Second row: alignments obtained using respective DRIFT-incorporated embeddings. Across slices, DRIFT yields stable and spatially coherent embryonic alignments, resulting in higher-fidelity 3D tissue reconstructions. Full results are available in Figs 22 and 23, available as supplementary data at *Bioinformatics* online.

We then applied DRIFT to reconstruct 3D tissue organization using the StereoME mouse embryo dataset, which provides spatial maps across early organogenesis. We aligned four consecutive E9.5 sections (Slice 1→2, 2→3, and 3→4) and reconstructed the resulting embeddings in 3D space. Visually, only the original scFoundation embeddings produce reasonably coherent alignments, whereas the other foundation models frequently yield inversions or rotations, highlighting the difficulty of this alignment task (Fig. 4, Figs 23 and 24, and Section 11, available as supplementary data at *Bioinformatics* online).

Incorporating DRIFT noticeably improves performance across all models in most scenarios, making 3D reconstructions more biologically consistent. Specifically, DRIFT consistently improves median alignment performance across most foundation models for both PCC and Kendall’s τ (Figs 22 and 23, available as supplementary data at *Bioinformatics* online). The largest and most consistent improvements are observed for scGPT and Geneformer, which exhibit increases of up to 4%–5% in PCC and over 7% in Kendall’s τ across multiple alignments. Loki demonstrates moderate but stable improvements while Nicheformer displays both the great improvements (up to 13% in PCC), correctly fixing one wrong alignment but shows small degradations in some other tasks where alignments with original alignments are already decent.

#### 3.2.3 Human small intestine dataset

**Figure 5:**
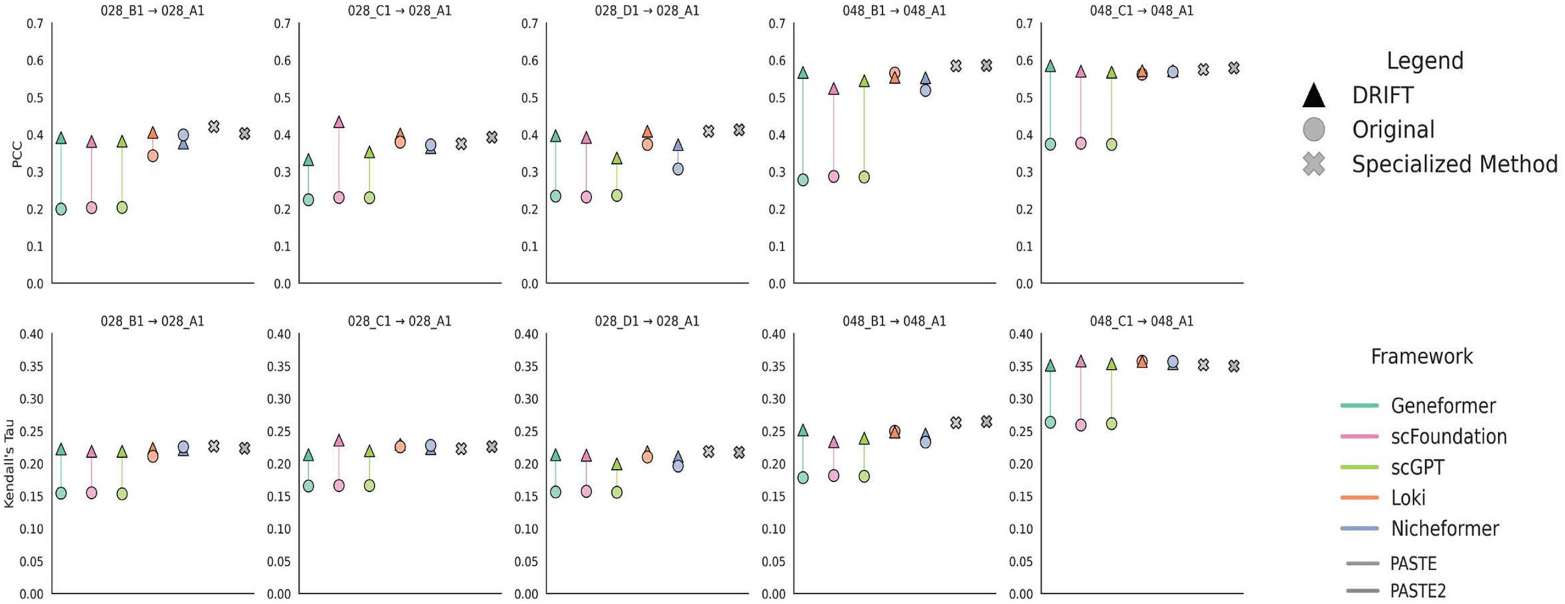
Human small intestine alignment results. Foundation models substantially improve alignment quality across human small intestine sections and become competitive with PASTE/PASTE2 when paired with DRIFT.

Finally, to further validate DRIFT’s alignment capability in real-world contexts, we aligned three sets of four adjacent human small intestine sections (10xHSI). Across all intestine slice pairings, DRIFT consistently improves alignment performance for most single-cell foundation models, as measured by both PCC and Kendall’s τ (Fig. 5 and Fig. 25, available as supplementary data at *Bioinformatics* online). scFoundation, scGPT, and Geneformer, exhibit the most substantial and consistent gains. These models show clear increases in median correlation under DRIFT. This suggests that their learned representations reinforces local neighborhood structures. In contrast, Loki demonstrates more modest improvements, with smaller but generally positive shifts in both PCC and Kendall’s τ. As for Nicheformer, while DRIFT improves performance in several pairings, the gains are less consistent and occasionally negligible. This trend suggests that ST-based foundation models may already encode aspects of spatial structure, thereby limiting the marginal benefit of additional diffusion for this specific dataset.

Altogether, DRIFT denoises spatial embeddings and reconstructs meaningful biological structure, yielding more accurate and coherent alignments.

### 3.3 DRIFT enhances clustering performance

Finally, we evaluated DRIFT’s performance on the unsupervised clustering task. We formulated the task as a zero-shot problem, with no re-training or fine-tuning. We used the pretrained weights of the foundation models (with and without DRIFT inputs) to obtain cell/spot embeddings and subsequently clustered them using the mclust algorithm (Scrucca *et al.* 2016). We evaluated all methods on the five datasets from the annotation task. The five datasets were 10xHPC (10x human prefrontal cortex), MERHH (MERFISH human heart), MERMPH (MERFISH mouse preoptic hypothalamus), MERMB (MERFISH mouse brain), and MERMBA (MERFISH mouse brain). The ground truth for clustering is cell-type labels annotated by experts. We clustered each slice independently and quantified the performance of the five foundation models and one specialized method (GraphST) with ARI as the evaluation metric.

As displayed in Table 2, DRIFT improves the zero-shot performance of all scRNA-seq foundation models. Quantitatively, the mean ARI scores rose substantially from 0.0778 to 0.2681 for Geneformer, 0.2558 to 0.5233 for scFoundation, and 0.2983 to 0.4470 for scGPT. These results further demonstrate that the diffused inputs generated by DRIFT better capture biologically meaningful structure, enabling foundation models to access richer spatial–molecular information in a zero-shot setting.

**Table 2:** Clustering results.^a^

	scRNA-seq foundation models						ST foundation models				
	Geneformer		scFoundation		scGPT		Loki		Nicheformer		GraphST
	Original	DRIFT	Original	DRIFT	Original	DRIFT	Original	DRIFT	Original	DRIFT	Original
10xHPC	0.0036	0.3238	0.3079	**0.4406**	0.3118	0.2314	0.3457	0.4022	0.2639	0.0696	0.3636
MERHH	0.0097	0.0194	0.2234	0.5739	0.3577	0.5451	0.4611	0.4358	0.0658	0.1580	**0.5994**
MERMPH	0.0234	0.4548	0.1179	**0.5493**	0.2073	0.5433	0.3059	0.4852	0.1039	0.4437	0.3309
MERMB	0.0090	0.0161	0.3075	**0.489**	0.4303	0.4515	0.4089	0.4375	0.4447	0.4417	0.4169
MERMBA	0.3435	0.5264	0.3224	0.5635	0.1848	0.4640	0.2529	**0.5650**	0.3082	0.5054	*

a The table lists the mean zero-shot ARI performance of every foundation model and its DRIFT-incorporated versions on each dataset. The bolded values represent the best-performing model for each dataset, while the underlined values represent the second best-performing model. These results show that the best-performing foundation models overall are DRIFT-incorporated scGPT, DRIFT-incorporated scFoundation, and DRIFT-incorporated Loki.

* —GraphST did not run on the MERMBA dataset.

We also see improved ARI for ST foundation models. The mean ARI scores rose from 0.3549 to 0.4651 for Loki, and from 0.2372 to 0.3236 for Nicheformer, confirming the effectiveness of DRIFT even on models trained with spatial data.

Interestingly, we observe that scFoundation, scGPT, and Loki models with DRIFT input outperformed GraphST’s mean ARI score of 0.4277, achieving mean ARIs of 0.5132, 0.4428, and 0.4401, respectively, on the four datasets for which GraphST could be successfully executed. Note that GraphST requires re-training on new samples, whereas foundation models with DRIFT input are run in a zero-shot setting. This result demonstrates that DRIFT effectively bridges the performance gap between general-purpose foundation models and specialized ST methods, allowing previously underperforming foundation models to achieve or exceed ST-specific benchmarks. In addition to the mean scores, we report the standard deviation across slices in Section 12, available as supplementary data at *Bioinformatics* online. We also observe that the performance of some of the foundation models can be further improved with few-shot training, as outlined in Section 13, available as supplementary data at *Bioinformatics* online.

## 4 Discussion and conclusion

We introduce DRIFT, a graph diffusion-based framework that integrates spatial context into the inputs of pretrained foundation models. DRIFT-incorporated foundation models improve performance across three ST tasks—annotation, alignment, and clustering—without requiring architectural changes or retraining.

DRIFT improves global transcriptomic structure and enhances marker-based cell identification and only introduces minimal distortion to spatial boundary organization, indicating a favorable balance between denoising and preservation of biologically meaningful structure. Although a diffusion time of *t* = 5 performs well across a wide range of datasets and tasks, this parameter can be tuned to balance smoothing strength against boundary fidelity. One potential direction to mitigate over-smoothing is to refine graph construction by incorporating gene expression similarity, enabling the pruning of edges between transcriptionally dissimilar cells. Such an approach could reduce unintended mixing across cell types; however, this remains an open area for future research.

Beyond its predictive performance, DRIFT is a lightweight, GPU-free module with relatively low computational requirements. To substantiate this, we benchmarked DRIFT’s diffusion runtime against the cost of fine-tuning and pretraining scGPT. Across all datasets and tissue slices, DRIFT’s longest recorded runtime was 485 s on a single CPU; comfortably under the 710 s required for fine-tuning scGPT on a single GPU. Both of these values are significantly lower than the scGPT pretraining cost reported by its authors, which needs 3 days of computation across 4 GPUs. This comparison highlights that DRIFT improves performance at lower costs, making it accessible in settings where GPU infrastructure is unavailable or limited. Its efficiency can be further improved by approximating the heat kernel using Chebyshev polynomials, reducing the time complexity from O($n^{3}$) to O(*ng*), where *n* is the number of cells/spots and *g* is the number of genes.

An interesting open research question concerns the variable performance improvement across different foundation models. We find that, with increasing levels of noise, the extent of improvement depends on how each foundation model encodes transcriptomic information. For rank-based models (Geneformer, Nicheformer, and Loki), which rely on gene ordering rather than absolute expression values, DRIFT-induced changes in gene ranking can improve greatly with increasing noise. We further observe that improvements are also influenced by training regimes in a lot of experiments. However, these behaviors are not uniform across datasets and tasks, and a definitive theoretical interaction mechanism remains open to further exploration.

DRIFT is a simple, effective, and accessible method for coupling pretrained scRNA-seq foundation models with ST data and performing diffusion-based denoising for ST foundational models. By combining pretrained embeddings with diffusion-enhanced information, DRIFT leverages existing models and extends them to a unified framework for spatially aware representations of tissues and developmental organization.

## Supplementary Material

btag259_Supplementary_Data

## Data Availability

All data supporting findings are publicly available from sources cited in the article.
