# Supplementary material for: Diffusion-based representation integration for foundation models improves spatial transcriptomics analysis
Source: Bioinformatics. 2026 Jul 7;42(Suppl 1):btag259. doi: 10.1093/bioinformatics/btag259 (PMC13340259; doi:10.1093/bioinformatics/btag259)
Supplement: btag259_Supplementary_Data [file btag259_supplementary_data.pdf]

Supplementary Information

Contents

S1 Graph Construction Comparison **2**

S2 Diffusion and Denoising Methods Comparison **2**

S3 Spatial Awareness Experiments **3**

S4 Geneformer Performance **4**

S5 Foundation Model Technical Explanation **4**

S6 Hyperparameter Tuning t **5**

S7 Hyperparameter Tuning Probes for Few-Shot Learning **5**

S8 Hyperparameter Tuning Specialized Methods **5**

S9 Annotation Accuracy Results **6**

S10 Boundary and Cell type analysis **6**

S11 Alignment Results **6**

S12 Clustering Standard Deviation Results **11**

S13 Few-Shot Clustering Results **12**

Figures **13**

S1 Graph Construction Comparison

To identify the optimal graph topology for DRIFT, we evaluated two construction techniques:

- **K-Nearest Neighbors** - For this graph construction technique, we connected each node to the k spatially closest nodes based on Euclidean distance. Then we converted all edges to undirected, ensuring that every node has a minimum degree of k (though some may have a higher degree). We refer to this construction as Kk, where k denotes the number of closest neighbors (e.g., K3 = 3-nearest neighbors).
- **Threshold-based Method** - For this technique, we determined the spatial Euclidean distance threshold that yields an *average* of n neighbors across the graph. Unlike the neighbor-based approach, this method allows the number of connections per node to vary significantly based on local tissue density. We refer to this construction as Tn, where n denotes the number of average neighbors (e.g., T3 = Threshold-based construction with an average of 3 neighbors).

	MERMB	MERMPH	10xHPC	MERHH	MERMBA
K3	<u>0.572</u>	**0.765**	**0.661**	<u>0.756</u>	<u>0.703</u>
K4	0.505	0.710	0.645	**0.777**	**0.728**
K5	**0.574**	0.615	<u>0.657</u>	0.749	0.632
K6	0.524	0.648	0.594	0.717	0.663
T3	0.456	0.702	0.640	0.680	0.582
T4	0.456	0.728	0.653	0.664	0.642
T5	0.469	<u>0.740</u>	0.642	0.699	0.702
T6	0.510	0.654	0.647	0.738	0.698

Table S1: F1 Scores of all graph construction methods per dataset. Bolded values represent the best graph construction choice, and underlined values represent the second-best.

	MERMB	MERMPH	10xHPC	MERHH	MERMBA
K3	<u>0.718</u>	**0.765**	<u>0.731</u>	0.844	<u>0.826</u>
K4	0.653	0.750	0.707	**0.859**	**0.853**
K5	**0.728**	0.693	0.723	<u>0.846</u>	0.768
K6	0.686	0.691	0.666	0.842	0.799
T3	0.624	0.692	0.722	0.782	0.707
T4	0.598	0.725	0.721	0.769	0.768
T5	0.659	<u>0.753</u>	0.705	0.805	<u>0.826</u>
T6	0.674	0.716	**0.733**	0.826	0.820

Table S2: Accuracy values of all graph construction methods per dataset. Bolded values represent the best graph construction choice, and underlined values represent the second-best.

As observed in Tables S1 and S2, the k-NN graph construction with k=3 (K3) consistently ranks among the top three best performing design choices across most datasets. Therefore, we can conclude that the graph construction we chose is robust across datasets. As a result, we use K3 as our graph construction technique of choice.

This behavior reflects a trade-off between preserving local structures and over-smoothing in the diffusion process. As k increases, additional edges expand the effective diffusion neighborhood, which can lead to over-smoothing beyond spatial borders, particularly in heterogeneous tissues or sharp boundaries. In contrast, smaller values of k (e.g., k = 3) better preserve local structure by restricting the diffusion effect. This allows the method to enhance the signal while reducing unintended mixing between distinct spatial regions. This trend is consistent across both regular grid-based and irregular single-cell resolutions, suggesting that performance is driven more by effective control of the interaction range than by matching the exact physical number of neighbors.

S2 Diffusion and Denoising Methods Comparison

We compared our heat kernel diffusion method to multiple graph diffusion techniques on the cell annotation task. The graph diffusion techniques are:

- **Random Walk Diffusion** - Let A be the adjacency matrix of the graph. The we calculate the degree matrix D as:

$$D_{ii} = \sum_{j} A_{ij} \tag{1}$$

We then calculate the transition matrix P for a random walk as:

$$P = D^{-1}A \tag{2}$$

Finally, given a the input gene expression matrix x_0, we calculate the diffused output matrix x_k as:

$$x_k = P^k x_o \tag{3}$$

Where k is a positive integer hyperparameter that determines the diffusion strength of the method. Higher k values result in smoothing across larger spatial distances. Since k can only take positive integer values, it is limited in how much it can be tuned relative to the heat kernel.

- **Mean** - For every node in mean diffusion, we take the gene expression vectors of the node and all its neighbors and return the mean expression as the diffused output.

- **Median** - For every node in median diffusion, we take the gene expression vectors of the node and all its neighbors and return the median expression as the diffused output.

- **Spectral Gaussian Filter** - The spectral Gaussian filter is a type of low-pass filter calculated through the eigenvalue decomposition. Let L be the normalized Laplacian of the graph.

$$L = U\Lambda U^{-1} = U\Lambda U^{\top} \tag{4}$$

We then apply the Gaussian filter $g(l)$ on the eigenvalues l. The filter is defined as:

$$g(l) = e^{\frac{-l^2}{2\sigma^2}} \tag{5}$$

where σ is the standard deviation of the Gaussian, which we set to 1.

- **Spectral Low-Pass Filter** - The spectral Low-Pass filter is a hard low-pass filter where any eigenvalues above a threshold are set to 0. Since we calculate the eigenvalues for a normalized Laplacian, the threshold is set at 0.5.

- **GraphST** - GraphST denoises data by aggregating spatial graph-based features, reconstructing gene expression, and utilizing contrastive learning to reinforce consistent spatial signals. [1], [2]

As seen in Figures S1-S14, the heat kernel shows an overall higher performance for top-performing foundation models, thus setting it as our choice of graph diffusion.

S3 Spatial Awareness Experiments

To evaluate the extent to which DRIFT-incorporated embeddings capture local spatial information, we conducted two complementary experiments. First, we extended the cell type annotation task by perturbing local spatial structure through randomized graph connectivity. Second, we assessed neighborhood awareness by formulating a neighboring-cell-type composition task, in which models predict the proportions of cell types within predefined spatial radii around each cell, similar to Schaar *et al.*[3]

In the first experiment, we assessed sensitivity to local spatial structure by randomizing graph connectivity prior to cell type annotation. As observed in Table S3, across datasets and foundation models, this perturbation led to a noticeable decline in annotation performance, indicating that accurate predictions depend on preserved spatial neighborhood information.

[1] Yahui Long, Kok Siong Ang, Mengwei Li, Kian Long Kelvin Chong, Raman Sethi, Chengwei Zhong, Hang Xu, Zhiwei Ong, Karishma Sachaphibulkij, Ao Chen, et al. Spatially informed clustering, integration, and deconvolution of spatial transcriptomics with graphst. *Nature Communications*, 14(1):1155, 2023

[2] Haiyue Wang, Peng Gao, Shaoqing Feng, and Xiaoke Ma. Denoising spatially resolved transcriptomics with consistency of heterogeneous spatial coordinates, transcription, and morphology. *Briefings in Bioinformatics*, 26(5):bbaf528, 2025

[3] Anna C Schaar, Alejandro Tejada-Lapuerta, Giovanni Palla, Robert Gutgesell, Lennard Halle, Mariia Minaeva, Larsen Vornholz, Leander Dony, Francesca Drummer, et al. Nicheformer: a foundation model for single-cell and spatial omics. *bioRxiv*, pages 2024–04, 2024

Table S3: Graph shuffling results in reduced cell-annotation model f1-score performance, indicating local spatial context is necessary for DRIFT. Shuffled Diffusion represents shuffling the neighbors in the graph and then performing diffusion. Higher values correspond to better performance.

		10xHPC	MERHH	MERMPH	MERMB	MERMBA
Geneformer	DRIFT	0.6085	0.671	0.6858	0.4286	0.4745
	Shuffled Diffusion	0.1315	0.059	0.0872	0.0718	0.0627
scFoundation	DRIFT	0.6017	0.6357	0.7178	0.4899	0.5829
	Shuffled Diffusion	0.1315	0.0872	0.059	0.0718	0.0627
scGPT	DRIFT	0.661	0.756	0.765	0.572	0.703
	Shuffled Diffusion	0.1133	0.045	0.06	0.066	0.042
Loki	DRIFT	0.6558	0.6731	0.7094	0.3794	0.6141
	Shuffled Diffusion	0.1351	0.0562	0.0702	0.0777	0.0421
Nicheformer	DRIFT	0.5659	0.6523	0.6754	0.4579	0.6068
	Shuffled Diffusion	0.1238	0.0329	0.0717	0.0707	0.0461

Table S4: Neighborhood composition prediction shows closer prediction to ground truth distributions with DRIFT. Original refers to performing the neighborhood task without any diffusion. Lower values correspond to better performance.

		10xHPC	MERHH	MERMPH	MERMB	MERMBA
Geneformer	DRIFT	0.049	0.0567	0.0577	0.583	0.0353
	Original	0.0851	0.0565	0.1029	0.0592	0.0675
scFoundation	DRIFT	0.023154	0.016641	0.033013	0.018502	0.026804
	Original	0.043738	0.027556	0.054934	0.031691	0.054698
scGPT	DRIFT	0.0634	0.0196	0.0403	0.0333	0.0402
	Original	0.0526	0.0302	0.0606	0.0413	0.0700
Loki	DRIFT	0.029762	0.013176	0.036412	0.027431	0.030464
	Original	0.049638	0.027323	0.056532	0.037651	0.056513
Nicheformer	DRIFT	0.038249	0.019111	0.036169	0.019549	0.032661
	Original	0.053462	0.034545	0.058315	0.0292	0.057233

In the second experiment, we evaluated neighborhood awareness by predicting the composition of cell types within predefined spatial radii around each cell, with performance measured using KL divergence between predicted and true neighborhood distributions. Specifically, given a true neighborhood composition $P = (p_1, \ldots, p_C)$ and a predicted composition $Q = (q_1, \ldots, q_C)$ over C cell types, we compute $\mathrm{KL}(P \| Q) = \sum_{c=1}^{C} p_c \log \frac{p_c}{q_c}$, where lower values indicate closer agreement between predicted and true neighborhood compositions. Across datasets and foundation models, DRIFT-incorporated embeddings consistently achieved lower KL divergence compared to baseline embeddings, indicating more accurate recovery of local cell type composition (Table S4).

Together, these two experiments demonstrate that DRIFT explicitly encodes local spatial context, with performance degrading under disrupted spatial structure but yielding more accurate neighborhood-level representations when spatial relationships are preserved.

S4 Geneformer Performance

We compared all three Geneformer models on the cell annotation task. As observed in Figures S9-S14, Geneformer V2 with 316 million parameters performed the best. Therefore, we use it as our choice of Geneformer.

S5 Foundation Model Technical Explanation

To ensure consistency with downstream foundation models trained on human gene identifiers, mouse datasets were mapped to their corresponding human orthologs using Ensembl BioMart. For each mouse gene, the associated human ortholog symbol and Ensembl gene identifier were retrieved. Genes without valid ortholog mappings were discarded, and duplicate mappings were resolved by retaining a single representative per mouse gene. The dataset was subsequently restricted to genes with valid orthologs, with human gene symbols used as primary identifiers.

The processed datasets were subsequently used as inputs to the DRIFT framework prior to integration with foundation models. Because each foundation model operates on a predefined gene vocabulary, genes not

represented in a model's native vocabulary were handled according to model-specific preprocessing strategies to ensure compatible input dimensionality. Different normalization and selection procedures were applied: Loki retained the top 50 most highly expressed genes per cell, scFoundation accommodated missing genes by assigning zero values, Nicheformer and Geneformer ranked genes within each cell based on expression magnitude, and scGPT discretized gene expression values into categorical bins. These procedures ensured consistency between the input data and each model's representation while preserving relative expression levels.

S6 Hyperparameter Tuning t

To test which value of t performed best, we ran all methods across all tasks with the following values of t - 10^{-5}, 10^{-4}, 10^{-3}, 10^{-2}, 10^{-1}, 1, 2, 5, 10, and 100. The performance of different values of the hyperparameter t in each task is illustrated in the following images:

- **Cell Annotation** - Figures S1-S10
- **Slice Alignment** - Tables S8 to S12
- **Clustering** - Figures S15-S19

Across all tasks, we note that $t \in \{1, 2, 5, 10\}$ tend to yield the best results.

For annotation and clustering, $t = 5$ performs well overall and is therefore chosen as the default value for these tasks.

For slice alignment, we select the hyperparameter that yields the highest Pearson correlation. Because this metric does not rely on labels, the tuning procedure can be applied in any setting.

S7 Hyperparameter Tuning Probes for Few-Shot Learning

To hyperparameter-tune the three-layered multilayer perceptron (MLP) foundation model probes that we train for few-shot learning, we employed Optuna for 10 trials. The range of values that we searched over is shown in Table S5.

Table S5: The range of values used for each hyperparameter during hyperparameter tuning.

**Hyperparameter**	**Values**
Hidden Size of Layer 1	10 - input embedding size
Hidden Size of Layer 2	10 - 256
Learning rate	10^{-5} - 10^{-1}
Weight Decay	10^{-6} - 10^{-3}
Dropout	0.1 - 0.6
Use Class Weights for Loss	True/False

S8 Hyperparameter Tuning Specialized Methods

5.1 STELLAR

For STELLAR, we also used Optuna for 10 trials to hyperparameter-tune the method. The range of values that we searched over is shown in Table S6.

Table S6: The range of values used for each hyperparameter during hyperparameter tuning.

**Hyperparameter**	**Values**
Learning rate	10^{-5} - 10^{-1}
Weight Decay	$5 * 10^{-3}$ - $5 * 10^{-1}$
Seed	1 - 20

5.2 PASTE and PASTE2

We tuned the hyperparameter $\alpha \in \{0.1 - 0.9\}$ with a step size of 0.1 for both PASTE and PASTE2, selecting the configuration that achieved the highest Pearson correlation.

5.3 GraphST

As mentioned in Section 3.3 of the main text, since clustering is an unsupervised task, we did not perform any hyperparameter tuning for the foundation models and GraphST.

S9 Annotation Accuracy Results

Figure S20 demonstrates DRIFT's improved annotation accuracy performance across foundation models and datasets. These performances are also compared to a state-of-the-art annotation method, STELLAR.

S10 Boundary and Cell type analysis

To investigate potential over-smoothing, we analyzed the effect of DRIFT on marker gene expression in a MERFISH mouse brain dataset containing 5,488 cells across 8 cell types.

To this end, we performed classification analysis in which each known cell-type-specific marker gene was treated as a classifier for its corresponding cell type. Classification performance was quantified using AUROC and AUPR scores.

Across evaluated markers, DRIFT improves AUROC and AUPR scores for 7 of 8 cases, with only one case (PVH / AR) showing a slight decrease, indicating that the effect is generally beneficial. These gains reflect improved biological signals rather than trivial smoothing. Notably, this improvement is also observed for relatively rare cell populations, including PVT ($\tilde{1}$.9% of cells) and V3 ($\tilde{5}$.3%), suggesting that DRIFT improves biological signals even in low-abundance cell types (see Table S7 below). We have revised the manuscript with the following description:

To better characterize marker gene localization, we compared marker-gene-based predicted positives against ground-truth biological layers using a top n% expression threshold, where n is set to the proportion of the corresponding cell type. This adaptive threshold ensures that the number of predicted positives is consistent with the expected abundance of each cell type. Under this definition, we analyze discordant high-expression patterns (high-expression cells outside the target layer).

Spatially, in the original data, discordant high-expression cells are scattered and unstructured. In contrast, after applying DRIFT, these patterns become more spatially organized, appearing outside the target layer, which could be due to spatial heterogeneity. Although discordant cells after DRIFT tend to concentrate near tissue boundaries, their total quantity is lower than in the original data, and the overall change to the biological boundaries is minimal.

Taken together, these results suggest that DRIFT improves global structures and enhances marker-based cell identification, including for low-abundance cell types. Moreover, the overall impact on boundary structure is minimal. Therefore, DRIFT can be interpreted as a general-purpose module that improves signal quality for downstream spatial transcriptomics analyses, while largely preserving underlying spatial organization.

Layer	Proportion	Marker Gene	AUROC		AUPR	
			Original	DRIFT	Original	DRIFT
fx	0.097303	OPALIN	0.639198	**0.897343**	0.240287	**0.670898**
BST	0.148324	OXTR	0.565651	**0.818383**	0.191477	**0.459615**
PVT	0.019315	SLC17A6	0.767906	**0.972697**	0.414594	**0.912735**
MPN	0.252915	ESR1	0.707390	**0.857403**	0.456537	**0.630623**
PV	0.060678	BDNF	0.557583	**0.704161**	0.075394	**0.106302**
V3	0.052660	AQP4	0.843687	**0.906097**	0.218222	**0.521618**
PVH	0.141399	AR	**0.487306**	0.463129	**0.131173**	0.120860
MPA	0.227406	NOS1	0.511272	**0.558698**	0.234857	**0.246305**

Table S7: Effect of DRIFT on marker-gene discrimination across cell types. For each metric, the higher value between Original and DRIFT is bolded. DRIFT improves AUROC and AUPR for 7 of 8 layers, with the largest gains observed in rare populations (e.g., PVT, V3), indicating enhanced signal recovery in low-abundance cell types.

S11 Alignment Results

Tables S8 to S12 show alignment results on mouse embryonic reconstruction for all foundation models with and without DRIFT.

Table S8: Geneformer's performance on mouse embryonic reconstruction

Experiment	Foundation model	Method	Pearson	Kendall
E95_S1 → E95_S2	Geneformer	Original	0.8243	0.3246
E95_S1 → E95_S2	Geneformer	Diffusion_5.0	0.8532	0.3456
E95_S1 → E95_S2	Geneformer	Diffusion_2.0	0.8478	0.3408
E95_S1 → E95_S2	Geneformer	Diffusion_100.0	0.8501	0.3433
E95_S1 → E95_S2	Geneformer	Diffusion_10.0	0.851	0.3441
E95_S1 → E95_S2	Geneformer	Diffusion_1.0	0.8468	0.3402
E95_S1 → E95_S2	Geneformer	Diffusion_0.1	0.8614	0.3497
E95_S1 → E95_S2	Geneformer	Diffusion_0.01	0.8607	0.3491
E95_S1 → E95_S2	Geneformer	Diffusion_0.001	0.8273	0.3251
E95_S1 → E95_S2	Geneformer	Diffusion_0.0001	0.8301	0.3253
E95_S1 → E95_S2	Geneformer	Diffusion_0.00001	0.831	0.3248
E95_S2 → E95_S3	Geneformer	Original	0.8185	0.3239
E95_S2 → E95_S3	Geneformer	Diffusion_5.0	0.8462	0.3366
E95_S2 → E95_S3	Geneformer	Diffusion_2.0	0.8462	0.3396
E95_S2 → E95_S3	Geneformer	Diffusion_100.0	0.8447	0.3402
E95_S2 → E95_S3	Geneformer	Diffusion_10.0	0.8477	0.3402
E95_S2 → E95_S3	Geneformer	Diffusion_1.0	0.8501	0.3423
E95_S2 → E95_S3	Geneformer	Diffusion_0.1	0.8517	0.3436
E95_S2 → E95_S3	Geneformer	Diffusion_0.01	0.8504	0.3431
E95_S2 → E95_S3	Geneformer	Diffusion_0.001	0.8561	0.3413
E95_S2 → E95_S3	Geneformer	Diffusion_0.0001	0.8525	0.3373
E95_S2 → E95_S3	Geneformer	Diffusion_0.00001	0.8113	0.3232
E95_S3 → E95_S4	Geneformer	Original	0.8138	0.3256
E95_S3 → E95_S4	Geneformer	Diffusion_5.0	0.849	0.3409
E95_S3 → E95_S4	Geneformer	Diffusion_2.0	0.7835	0.329
E95_S3 → E95_S4	Geneformer	Diffusion_100.0	0.8217	0.3348
E95_S3 → E95_S4	Geneformer	Diffusion_10.0	0.8497	0.3416
E95_S3 → E95_S4	Geneformer	Diffusion_1.0	0.8176	0.3341
E95_S3 → E95_S4	Geneformer	Diffusion_0.1	0.8728	0.3546
E95_S3 → E95_S4	Geneformer	Diffusion_0.01	0.8619	0.3513
E95_S3 → E95_S4	Geneformer	Diffusion_0.001	0.8049	0.3321
E95_S3 → E95_S4	Geneformer	Diffusion_0.0001	0.8129	0.3252
E95_S3 → E95_S4	Geneformer	Diffusion_0.00001	0.8167	0.3264

Table S9: scFoundation's performance on mouse embryonic reconstruction

Experiment	Foundation model	Method	Pearson	Kendall
E95_S1 → E95_S2	scfoundation	Original	0.869	0.3526
E95_S1 → E95_S2	scfoundation	Diffusion_5.0	0.8762	0.3567
E95_S1 → E95_S2	scfoundation	Diffusion_2.0	0.8408	0.3247
E95_S1 → E95_S2	scfoundation	Diffusion_100.0	0.8602	0.3503
E95_S1 → E95_S2	scfoundation	Diffusion_10.0	0.8753	0.3586
E95_S1 → E95_S2	scfoundation	Diffusion_1.0	0.864	0.3528
E95_S1 → E95_S2	scfoundation	Diffusion_0.1	0.8796	0.3603
E95_S1 → E95_S2	scfoundation	Diffusion_0.01	0.8724	0.3567
E95_S1 → E95_S2	scfoundation	Diffusion_0.001	0.8801	0.3595
E95_S1 → E95_S2	scfoundation	Diffusion_0.0001	0.8804	0.3597
E95_S1 → E95_S2	scfoundation	Diffusion_0.00001	0.8804	0.3599
E95_S2 → E95_S3	scfoundation	Original	0.8523	0.3438
E95_S2 → E95_S3	scfoundation	Diffusion_5.0	0.8467	0.3415
E95_S2 → E95_S3	scfoundation	Diffusion_2.0	0.8492	0.3416
E95_S2 → E95_S3	scfoundation	Diffusion_100.0	0.8429	0.3357
E95_S2 → E95_S3	scfoundation	Diffusion_10.0	0.8492	0.339
E95_S2 → E95_S3	scfoundation	Diffusion_1.0	0.8477	0.3416
E95_S2 → E95_S3	scfoundation	Diffusion_0.1	0.8458	0.3412
E95_S2 → E95_S3	scfoundation	Diffusion_0.01	0.8473	0.3407
E95_S2 → E95_S3	scfoundation	Diffusion_0.001	0.8475	0.3421
E95_S2 → E95_S3	scfoundation	Diffusion_0.0001	0.8467	0.3422
E95_S2 → E95_S3	scfoundation	Diffusion_0.00001	0.8469	0.3423
E95_S3 → E95_S4	scfoundation	Original	0.8496	0.3426
E95_S3 → E95_S4	scfoundation	Diffusion_5.0	0.8472	0.3452
E95_S3 → E95_S4	scfoundation	Diffusion_2.0	0.8593	0.3505
E95_S3 → E95_S4	scfoundation	Diffusion_100.0	0.849	0.3432
E95_S3 → E95_S4	scfoundation	Diffusion_10.0	0.8258	0.338
E95_S3 → E95_S4	scfoundation	Diffusion_1.0	0.864	0.3522
E95_S3 → E95_S4	scfoundation	Diffusion_0.1	0.8153	0.3276
E95_S3 → E95_S4	scfoundation	Diffusion_0.01	0.8283	0.3379
E95_S3 → E95_S4	scfoundation	Diffusion_0.001	0.8498	0.3422
E95_S3 → E95_S4	scfoundation	Diffusion_0.0001	0.849	0.3417
E95_S3 → E95_S4	scfoundation	Diffusion_0.00001	0.8492	0.3417

Table S10: scGPT's performance on mouse embryonic reconstruction

Experiment	Foundation model	Method	LTARI	Pearson	Kendall
E95_S1 → E95_S2	scgpt	Original	0.0926	0.8493	0.3347
E95_S1 → E95_S2	scgpt	Diffusion_5.0	0.223	0.8783	0.3592
E95_S1 → E95_S2	scgpt	Diffusion_2.0	0.2316	0.8775	0.3601
E95_S1 → E95_S2	scgpt	Diffusion_100.0	0.254	0.8799	0.3604
E95_S1 → E95_S2	scgpt	Diffusion_10.0	0.23	0.8781	0.3596
E95_S1 → E95_S2	scgpt	Diffusion_1.0	0.214	0.8776	0.3581
E95_S1 → E95_S2	scgpt	Diffusion_0.1	0.2144	0.8767	0.3569
E95_S1 → E95_S2	scgpt	Diffusion_0.01	0.218	0.8771	0.3585
E95_S1 → E95_S2	scgpt	Diffusion_0.001	0.2126	0.8783	0.3581
E95_S1 → E95_S2	scgpt	Diffusion_0.0001	0.2478	0.8813	0.3603
E95_S1 → E95_S2	scgpt	Diffusion_0.00001	0.1997	0.8679	0.3545
E95_S2 → E95_S3	scgpt	Original	0.1668	0.8471	0.339
E95_S2 → E95_S3	scgpt	Diffusion_5.0	0.195	0.8518	0.3435
E95_S2 → E95_S3	scgpt	Diffusion_2.0	0.2013	0.8517	0.3437
E95_S2 → E95_S3	scgpt	Diffusion_100.0	0.1958	0.8476	0.3415
E95_S2 → E95_S3	scgpt	Diffusion_10.0	0.1925	0.8493	0.3428
E95_S2 → E95_S3	scgpt	Diffusion_1.0	0.2	0.8523	0.3447
E95_S2 → E95_S3	scgpt	Diffusion_0.1	0.1961	0.8526	0.3441
E95_S2 → E95_S3	scgpt	Diffusion_0.01	0.1929	0.8504	0.3428
E95_S2 → E95_S3	scgpt	Diffusion_0.001	0.1842	0.85	0.3431
E95_S2 → E95_S3	scgpt	Diffusion_0.0001	0.1907	0.8518	0.3448
E95_S2 → E95_S3	scgpt	Diffusion_0.00001	0.1892	0.8506	0.3427
E95_S3 → E95_S4	scgpt	Original	0.08	0.7872	0.3255
E95_S3 → E95_S4	scgpt	Diffusion_5.0	0.1271	0.8367	0.3393
E95_S3 → E95_S4	scgpt	Diffusion_2.0	0.2429	0.8716	0.3539
E95_S3 → E95_S4	scgpt	Diffusion_100.0	0.1382	0.7928	0.3288
E95_S3 → E95_S4	scgpt	Diffusion_10.0	0.1718	0.8538	0.3481
E95_S3 → E95_S4	scgpt	Diffusion_1.0	0.2548	0.8783	0.3545
E95_S3 → E95_S4	scgpt	Diffusion_0.1	0.1418	0.8486	0.3436
E95_S3 → E95_S4	scgpt	Diffusion_0.01	0.1077	0.8289	0.3261
E95_S3 → E95_S4	scgpt	Diffusion_0.001	0.1228	0.8317	0.3256
E95_S3 → E95_S4	scgpt	Diffusion_0.0001	0.1511	0.8552	0.3422
E95_S3 → E95_S4	scgpt	Diffusion_0.00001	0.1325	0.8342	0.3394

Table S11: Loki's performance on mouse embryonic reconstruction

Experiment	Foundation model	Method	Pearson	Kendall
E95_S1 → E95_S2	Loki	Original	0.832	0.3282
E95_S1 → E95_S2	Loki	Diffusion_5.0	0.8722	0.3576
E95_S1 → E95_S2	Loki	Diffusion_2.0	0.864	0.3526
E95_S1 → E95_S2	Loki	Diffusion_100.0	0.867	0.3534
E95_S1 → E95_S2	Loki	Diffusion_10.0	0.8754	0.3581
E95_S1 → E95_S2	Loki	Diffusion_1.0	0.8696	0.3546
E95_S1 → E95_S2	Loki	Diffusion_0.1	0.8326	0.319
E95_S1 → E95_S2	Loki	Diffusion_0.01	0.8424	0.3296
E95_S1 → E95_S2	Loki	Diffusion_0.001	0.8411	0.3305
E95_S1 → E95_S2	Loki	Diffusion_0.0001	0.8412	0.3305
E95_S1 → E95_S2	Loki	Diffusion_0.00001	0.8413	0.3305
E95_S2 → E95_S3	Loki	Original	0.843	0.3365
E95_S2 → E95_S3	Loki	Diffusion_5.0	0.8438	0.3387
E95_S2 → E95_S3	Loki	Diffusion_2.0	0.8434	0.3229
E95_S2 → E95_S3	Loki	Diffusion_100.0	0.8494	0.3334
E95_S2 → E95_S3	Loki	Diffusion_10.0	0.8548	0.3378
E95_S2 → E95_S3	Loki	Diffusion_1.0	0.8433	0.337
E95_S3 → E95_S4	Loki	Original	0.8826	0.359
E95_S3 → E95_S4	Loki	Diffusion_5.0	0.8795	0.3564
E95_S3 → E95_S4	Loki	Diffusion_2.0	0.8612	0.3509
E95_S3 → E95_S4	Loki	Diffusion_100.0	0.8465	0.3464
E95_S3 → E95_S4	Loki	Diffusion_10.0	0.8765	0.3549
E95_S3 → E95_S4	Loki	Diffusion_1.0	0.8584	0.3481
E95_S3 → E95_S4	Loki	Diffusion_0.1	0.8775	0.3572
E95_S3 → E95_S4	Loki	Diffusion_0.01	0.8855	0.3601
E95_S3 → E95_S4	Loki	Diffusion_0.001	0.8857	0.3625
E95_S3 → E95_S4	Loki	Diffusion_0.00001	0.8864	0.3622

Table S12: Nicheformer's performance on mouse embryonic reconstruction

Experiment	Foundation model	Method	Pearson	Kendall
E95_S1 → E95_S2	Nicheformer	Original	0.8785	0.359
E95_S1 → E95_S2	Nicheformer	Diffusion_5.0	0.8518	0.3396
E95_S1 → E95_S2	Nicheformer	Diffusion_2.0	0.8458	0.339
E95_S1 → E95_S2	Nicheformer	Diffusion_100.0	0.838	0.3264
E95_S1 → E95_S2	Nicheformer	Diffusion_10.0	0.8514	0.3395
E95_S1 → E95_S2	Nicheformer	Diffusion_1.0	0.8463	0.3395
E95_S1 → E95_S2	Nicheformer	Diffusion_0.1	0.8512	0.3377
E95_S1 → E95_S2	Nicheformer	Diffusion_0.01	0.85	0.3387
E95_S1 → E95_S2	Nicheformer	Diffusion_0.001	0.8396	0.3356
E95_S1 → E95_S2	Nicheformer	Diffusion_0.0001	0.8497	0.3429
E95_S1 → E95_S2	Nicheformer	Diffusion_0.00001	0.8526	0.3402
E95_S2 → E95_S3	Nicheformer	Original	0.8497	0.3414
E95_S2 → E95_S3	Nicheformer	Diffusion_5.0	0.8412	0.3355
E95_S2 → E95_S3	Nicheformer	Diffusion_2.0	0.8438	0.3376
E95_S2 → E95_S3	Nicheformer	Diffusion_100.0	0.8357	0.3394
E95_S2 → E95_S3	Nicheformer	Diffusion_10.0	0.8386	0.3352
E95_S2 → E95_S3	Nicheformer	Diffusion_1.0	0.8447	0.339
E95_S2 → E95_S3	Nicheformer	Diffusion_0.1	0.8453	0.3389
E95_S2 → E95_S3	Nicheformer	Diffusion_0.01	0.8431	0.3388
E95_S2 → E95_S3	Nicheformer	Diffusion_0.001	0.8259	0.3322
E95_S2 → E95_S3	Nicheformer	Diffusion_0.0001	0.8117	0.3191
E95_S2 → E95_S3	Nicheformer	Diffusion_0.00001	0.849	0.3401
E95_S3 → E95_S4	Nicheformer	Original	0.7857	0.3255
E95_S3 → E95_S4	Nicheformer	Diffusion_5.0	0.8896	0.3621
E95_S3 → E95_S4	Nicheformer	Diffusion_2.0	0.8823	0.3591
E95_S3 → E95_S4	Nicheformer	Diffusion_100.0	0.8686	0.3485
E95_S3 → E95_S4	Nicheformer	Diffusion_10.0	0.8851	0.3605
E95_S3 → E95_S4	Nicheformer	Diffusion_1.0	0.8728	0.3568
E95_S3 → E95_S4	Nicheformer	Diffusion_0.1	0.8767	0.357
E95_S3 → E95_S4	Nicheformer	Diffusion_0.01	0.8721	0.3561
E95_S3 → E95_S4	Nicheformer	Diffusion_0.001	0.8749	0.3562
E95_S3 → E95_S4	Nicheformer	Diffusion_0.0001	0.7814	0.326
E95_S3 → E95_S4	Nicheformer	Diffusion_0.00001	0.7656	0.3213

S12 Clustering Standard Deviation Results

In the main paper we report the mean clustering results across the slices. In Tables S13 and S14, we also report the standard deviation results for completeness.

	scRNA-seq foundation models					
	Geneformer		scFoundation		scGPT	
	Original	DRIFT	Original	DRIFT	Original	DRIFT
10xHPC	0.0036 ± 0.0058	0.3238 ± 0.0763	0.3079 ± 0.0886	$\mathbf{0.4406 \pm 0.0885}$	0.3118 ± 0.0855	0.2314 ± 0.0969
MERHH	0.0097 ± 0.0001	0.0194 ± 0.0046	0.2234 ± 0.0097	0.5739 ± 0.0759	0.3577 ± 0.0160	0.5451 ± 0.0301
MERMPH	0.0234 ± 0.0107	0.4548 ± 0.0808	0.1179 ± 0.0156	$\mathbf{0.5493 \pm 0.0438}$	0.2073 ± 0.0626	$\underline{0.5433 \pm 0.0753}$
MERMB	0.0090 ± 0.0117	0.0161 ± 0.0085	0.3075 ± 0.1241	$\mathbf{0.4893 \pm 0.0716}$	0.4303 ± 0.1199	$\underline{0.4515 \pm 0.1053}$
MERMBA	0.3435 ± 0.1514	0.5264 ± 0.1271	0.3224 ± 0.1310	$\underline{0.5635 \pm 0.1508}$	0.1848 ± 0.0830	0.4640 ± 0.0866

Table S13: **Clustering Standard Deviation Results Part 1 -** The table lists the mean and standard deviation of ARI performance of all scRNA-seq foundation models. Bolded values represent the best performance, while underlined values represent the second best across all models.

	ST foundation models				
	Loki		Nicheformer		GraphST
	Original	DRIFT	Original	DRIFT	Original
10xHPC	0.3457 ± 0.0622	$\underline{0.4022 \pm 0.0563}$	0.2639 ± 0.1415	0.0696 ± 0.0345	0.3636 ± 0.1035
MERHH	0.4611 ± 0.0099	0.4358 ± 0.0210	0.0658 ± 0.0019	0.1580 ± 0.0144	$\mathbf{0.5994 \pm 0.0040}$
MERMPH	0.3059 ± 0.0876	0.4852 ± 0.0446	0.1039 ± 0.0071	0.4437 ± 0.0722	0.3309 ± 0.0665
MERMB	0.4089 ± 0.1027	0.4375 ± 0.1028	0.4447 ± 0.1152	0.4417 ± 0.0720	0.4169 ± 0.1161
MERMBA	0.2529 ± 0.1449	$\mathbf{0.5650 \pm 0.1375}$	0.3082 ± 0.1467	0.5054 ± 0.1463	*

Table S14: **Clustering Standard Deviation Results Part 2** - The table lists the mean and standard deviation of ARI performance of all ST foundation models and GraphST. Bolded values represent the best performance, while underlined values represent the second best across all models.

S13 Few-Shot Clustering Results

Table S15 illustrates the dataset-specific few-shot clustering performance of scGPT and scFoundation and their DRIFT-enhanced versions after fine-tuning on only one train dataset. For few-shot learning, we perform a task similar to GraphST, where the model aims to reconstruct the input gene-expression profile. DRIFT-incorporated scFoundation's mean ARI performance shows almost no improvement from 0.523 in the zero-shot setting to 0.525 in the few-shot setting. However, DRIFT-incorporated scGPT displays major improvement with fine-tuning, with its mean ARI improving from 0.447 in the zero-shot setting to 0.5556 in the few-shot setting.

Table S15: **Clustering Results** - The table lists the mean few-shot ARI performance of scGPT, scFoundation, and their DRIFT-incorporated versions on every dataset. These results show that few-shot training can improve the performance of some foundation models.

	Original scGPT	DRIFT scGPT	Original scFoundation	DRIFT scFoundation
10xHPC	0.3193	0.5904	0.3103	0.4829
MERHH	0.6207	0.5779	0.4244	0.5358
MERMPH	0.2967	0.5076	0.1712	0.5190
MERMB	0.4301	0.4118	0.3582	0.4858
MERMBA	0.3511	0.6901	0.3972	0.6014

Figures

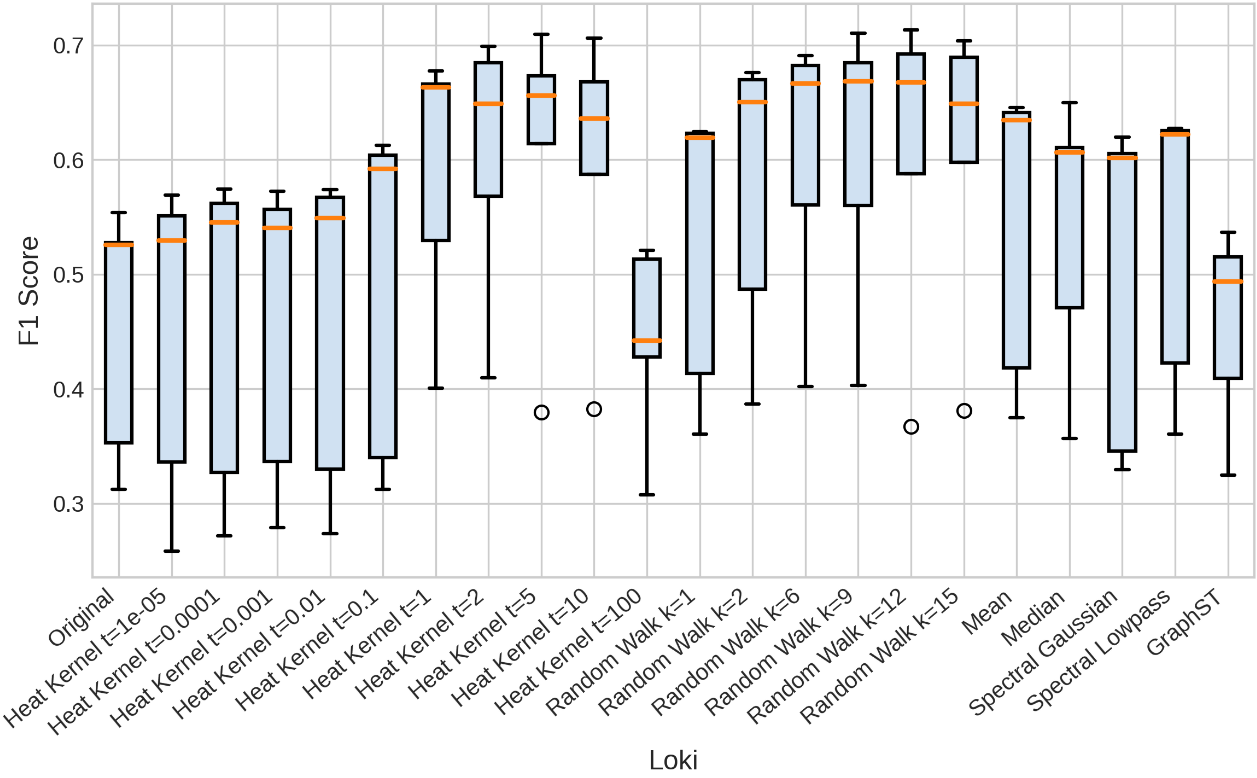

Figure S1: F1-scores of different diffusion methods for Loki over all datasets in the cell annotation task.

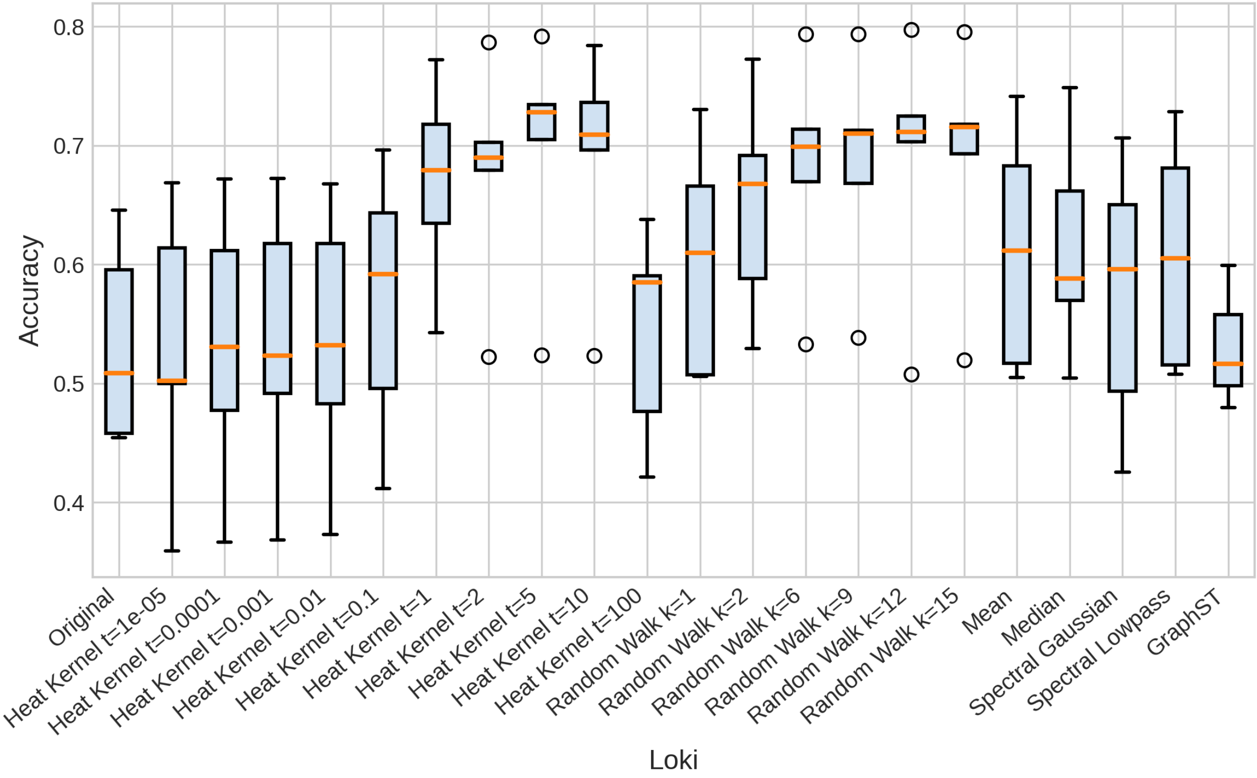

Figure S2: Accuracy of different diffusion methods for Loki over all datasets in the cell annotation task.

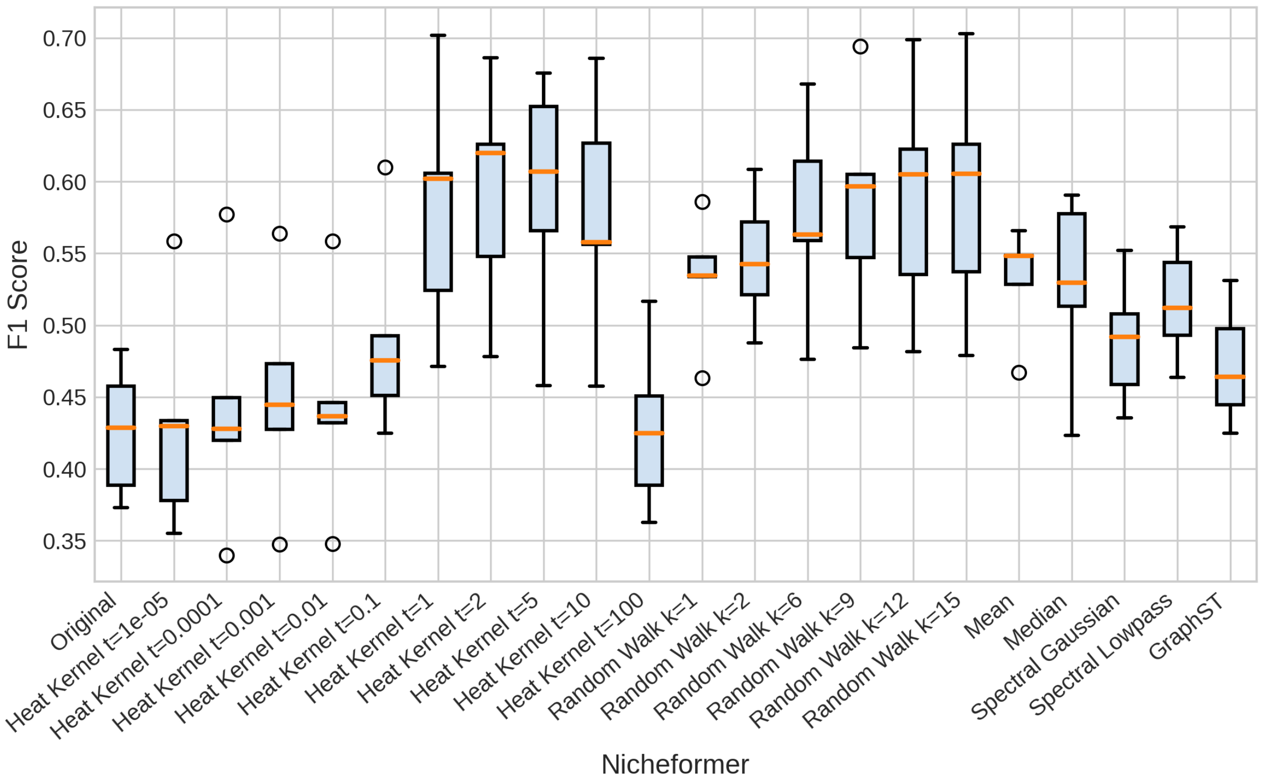

Figure S3: F1-scores of different diffusion methods for Nicheformer over all datasets in the cell annotation task.

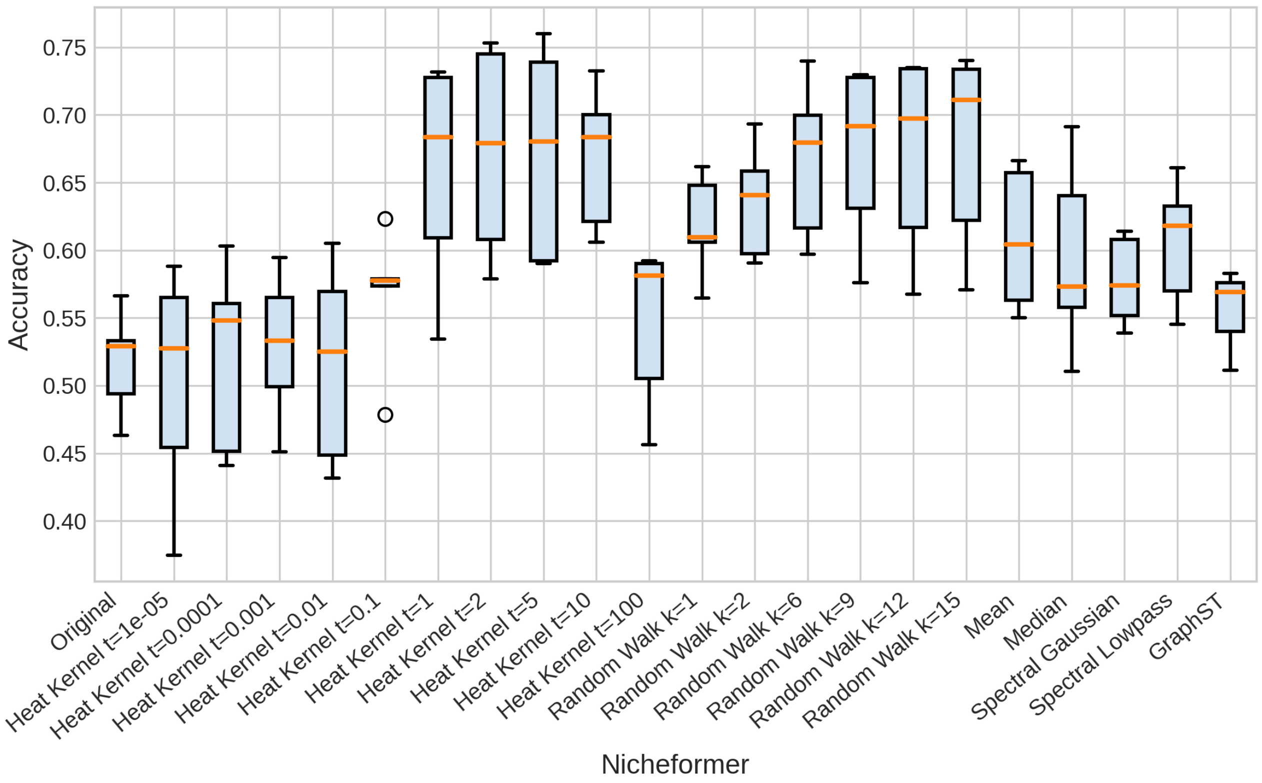

Figure S4: Accuracy of different diffusion methods for Nicheformer over all datasets in the cell annotation task.

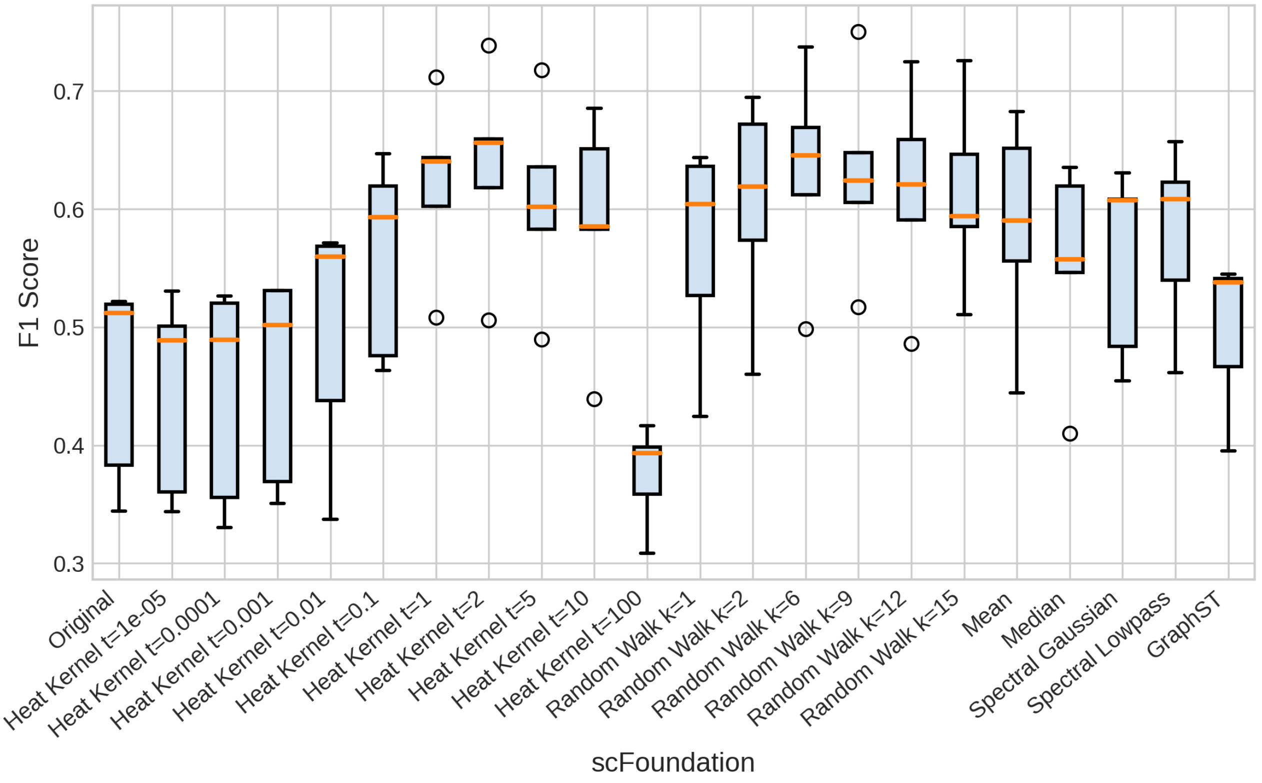

Figure S5: F1-scores of different diffusion methods for scFoundation over all datasets in the cell annotation task.

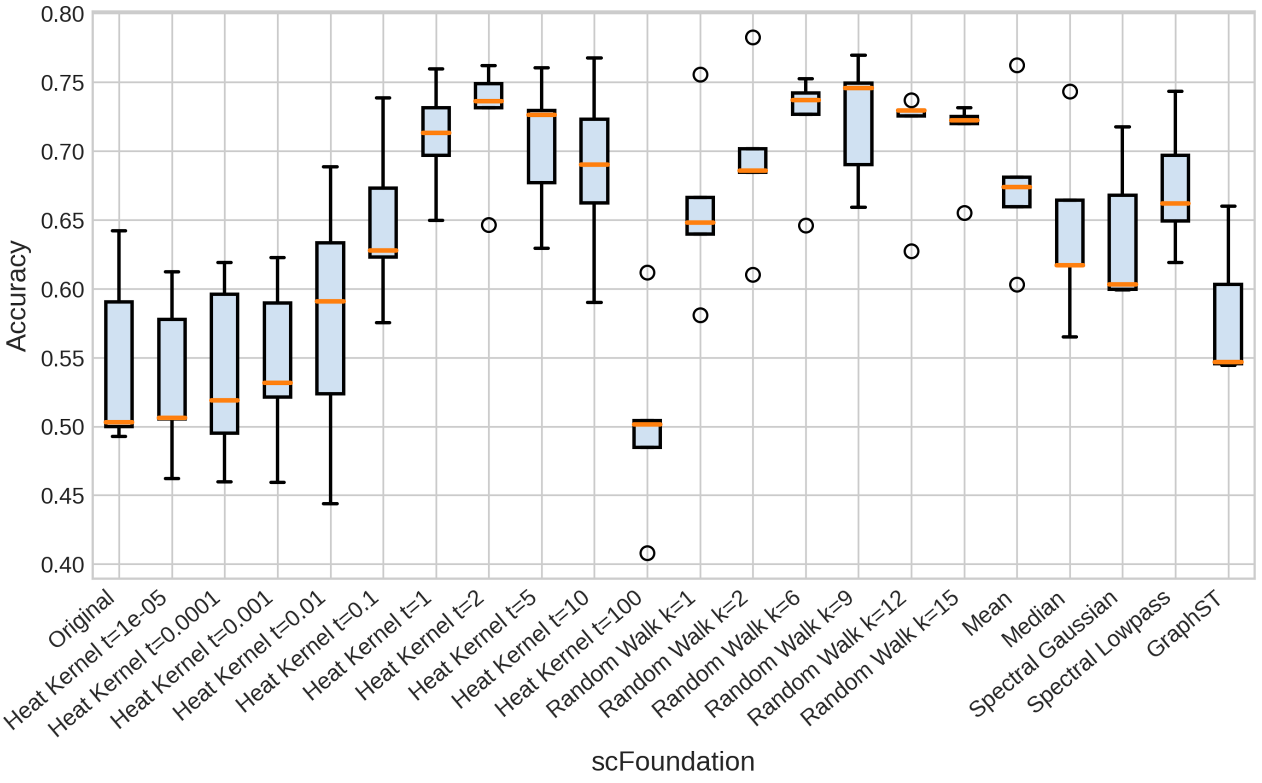

Figure S6: Accuracy of different diffusion methods for scFoundation over all datasets in the cell annotation task.

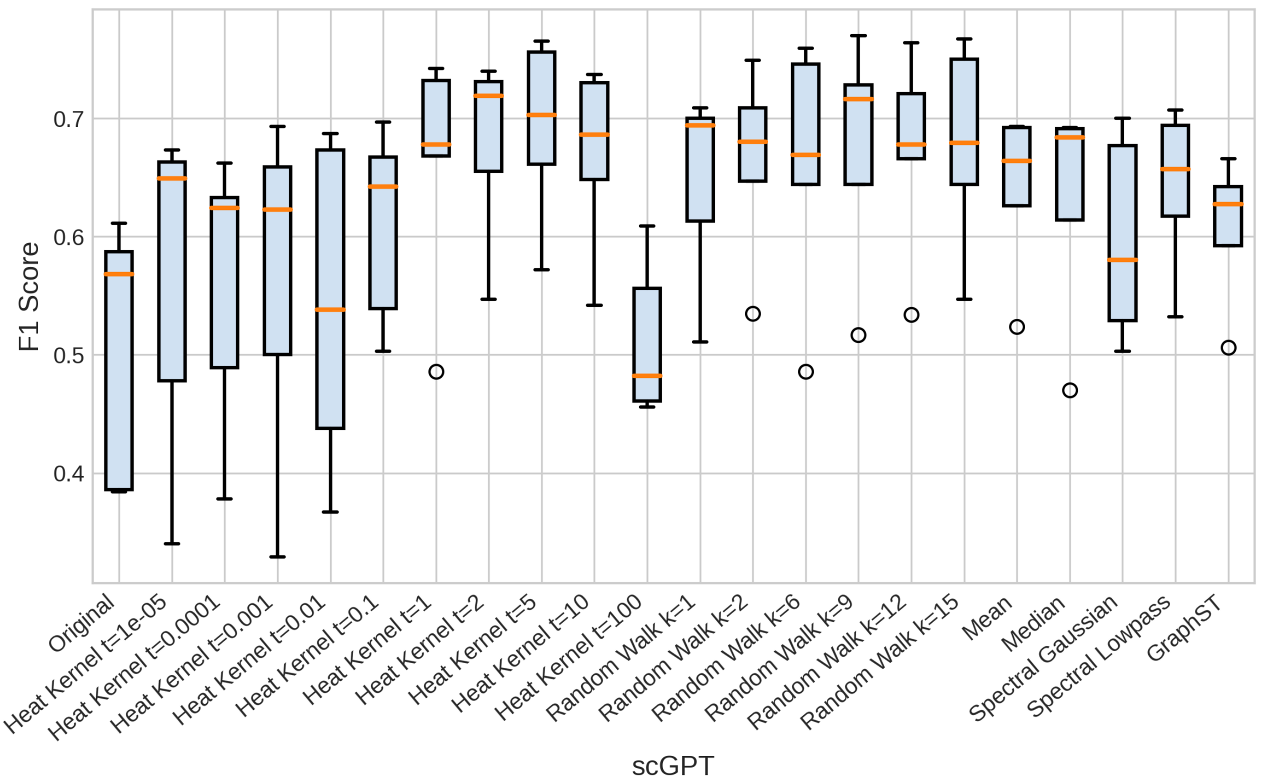

Figure S7: F1-scores of different diffusion methods for scGPT over all datasets in the cell annotation task.

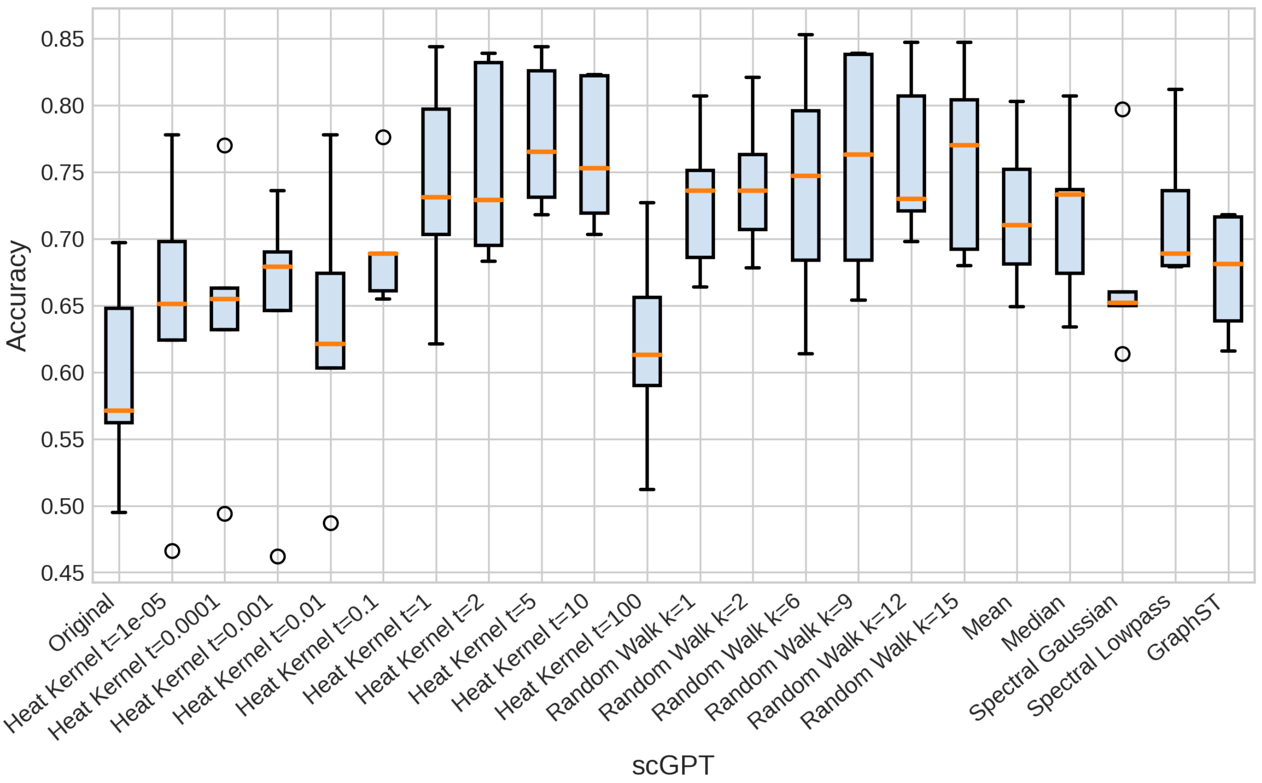

Figure S8: Accuracy of different diffusion methods for scGPT over all datasets in the cell annotation task.

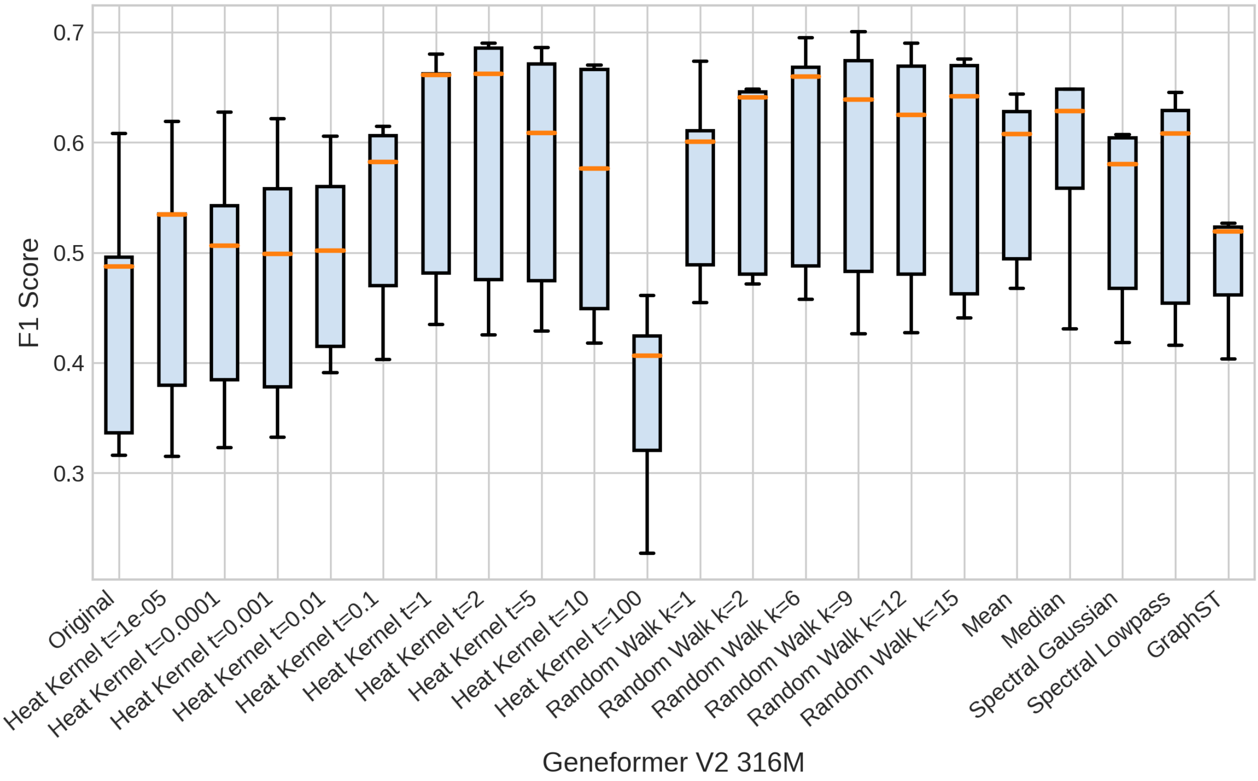

Figure S9: F1-scores of different diffusion methods for Geneformer V2 with 316 million parameters over all datasets in the cell annotation task.

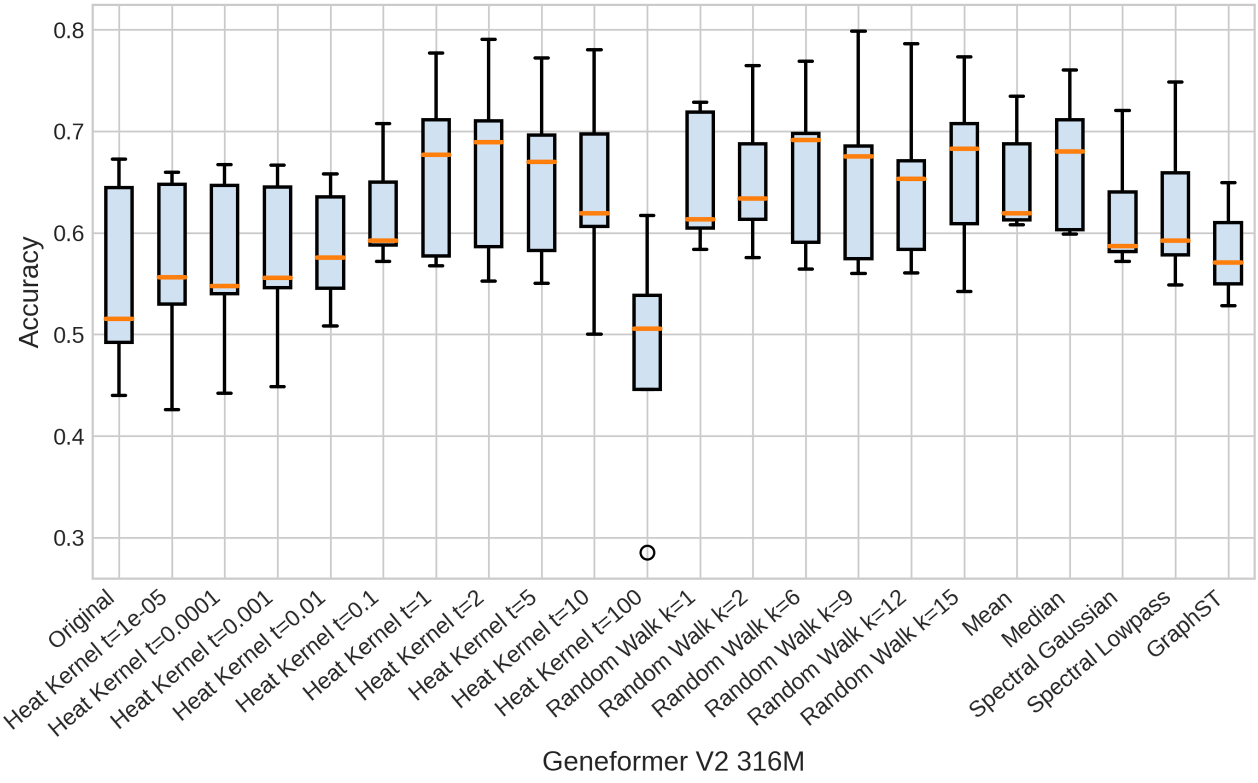

Figure S10: Accuracy of different diffusion methods for Geneformer V2 with 316 million parameters over all datasets in the cell annotation task.

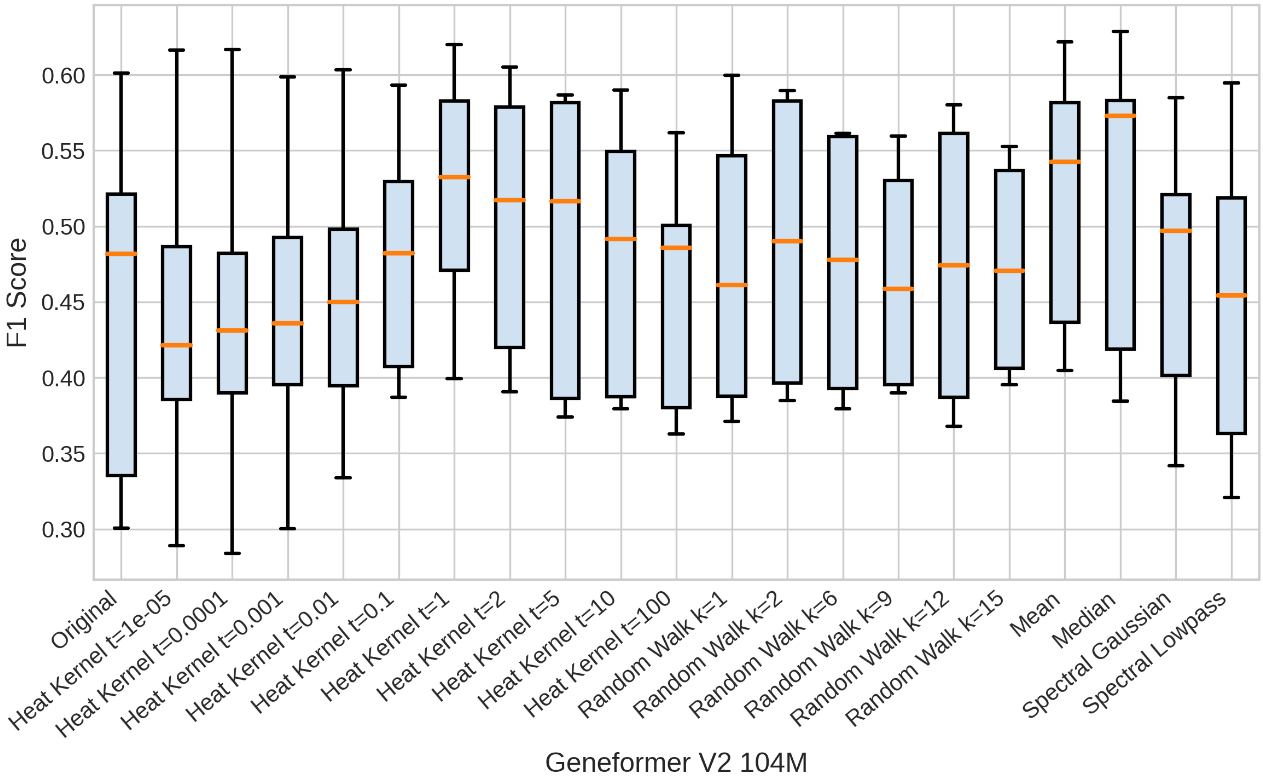

Figure S11: F1-scores of different diffusion methods for Geneformer V2 with 104 million parameters over all datasets in the cell annotation task.

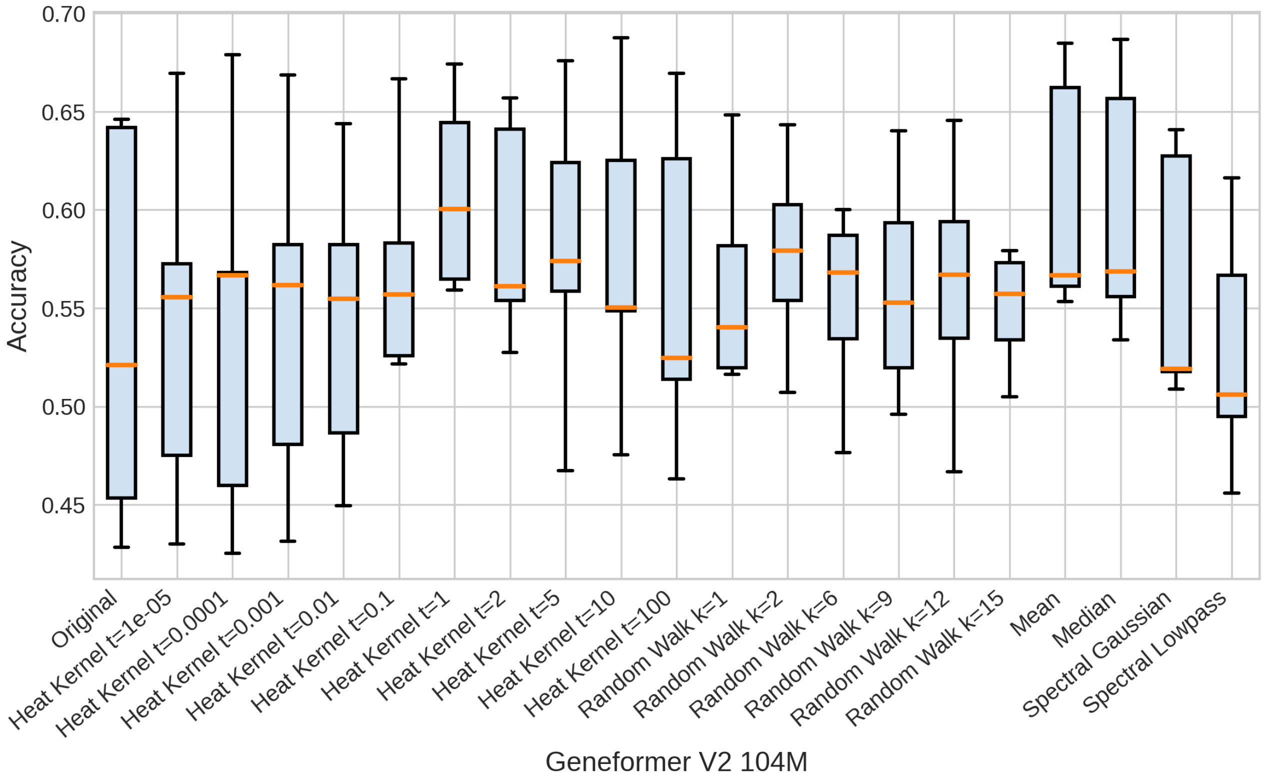

Figure S12: Accuracy of different diffusion methods for Geneformer V2 with 104 million parameters over all datasets in the cell annotation task.

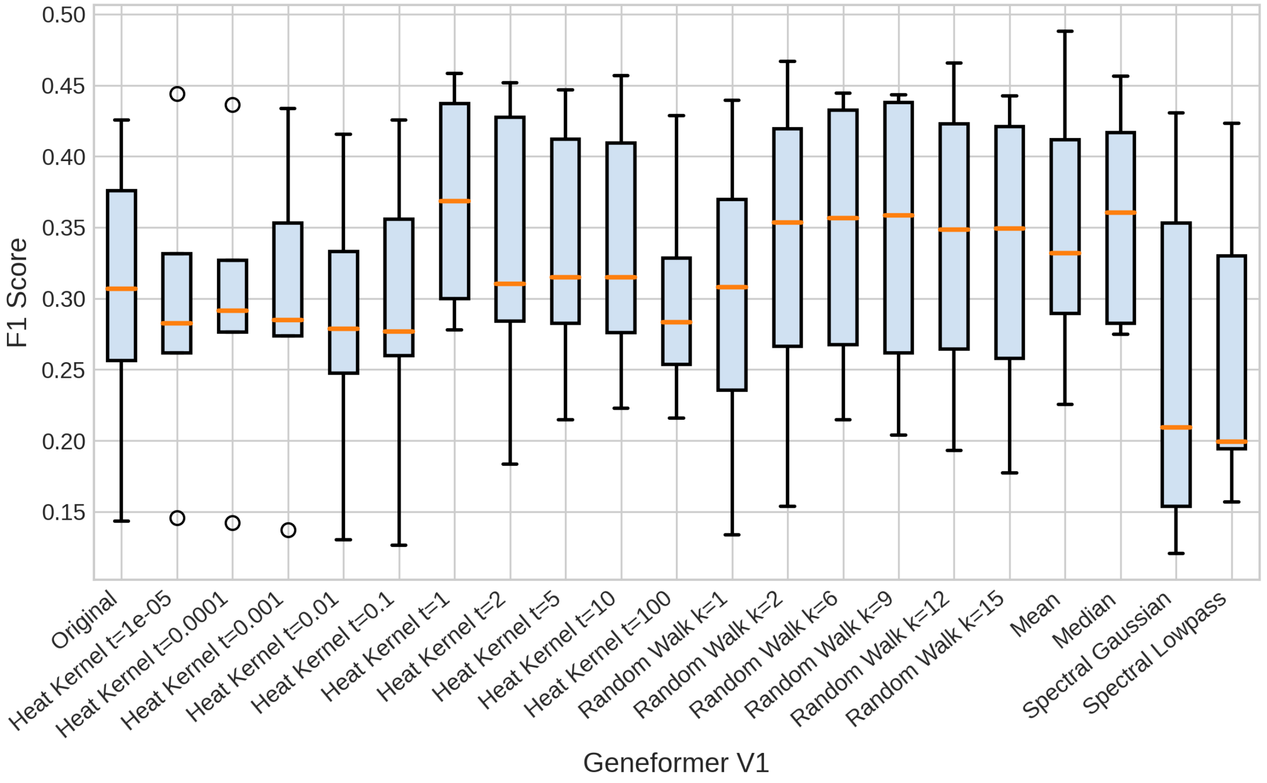

Figure S13: F1-scores of different diffusion methods for Geneformer V1 over all datasets in the cell annotation task.

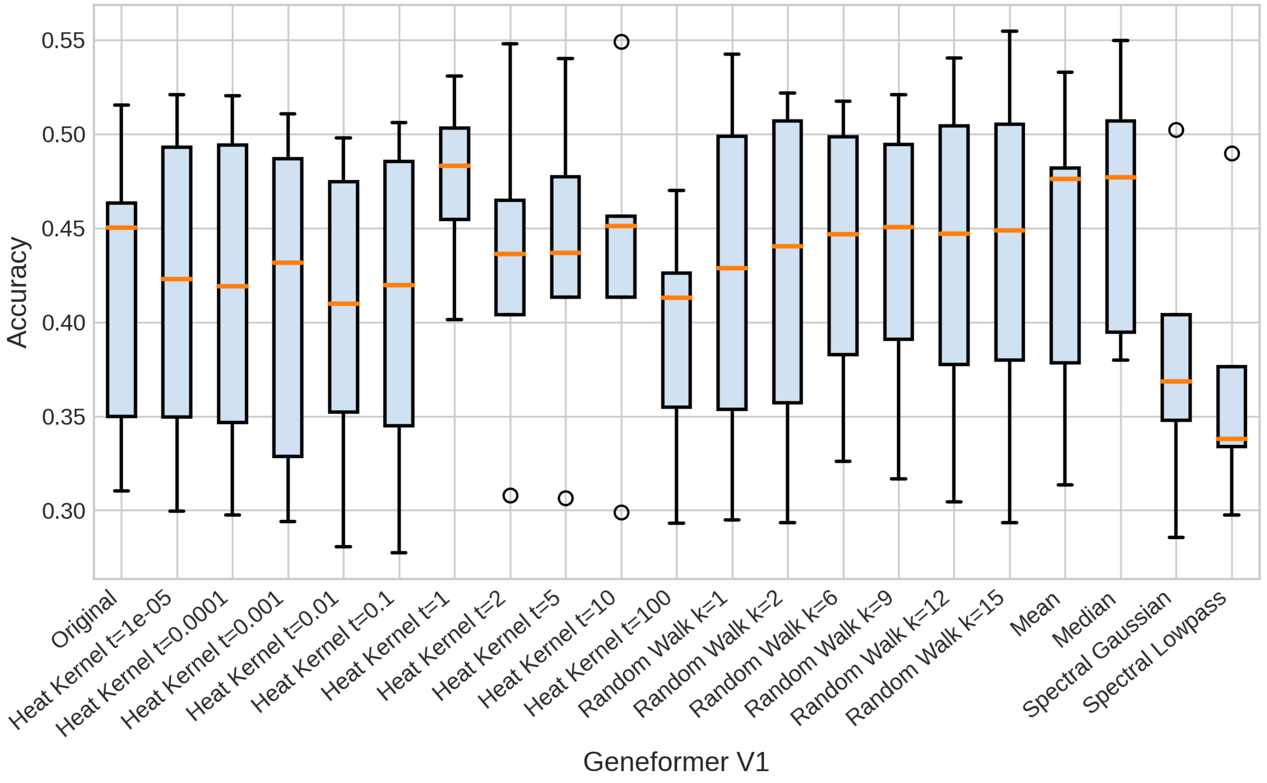

Figure S14: Accuracy of different diffusion methods for Geneformer V1 over all datasets in the cell annotation task.

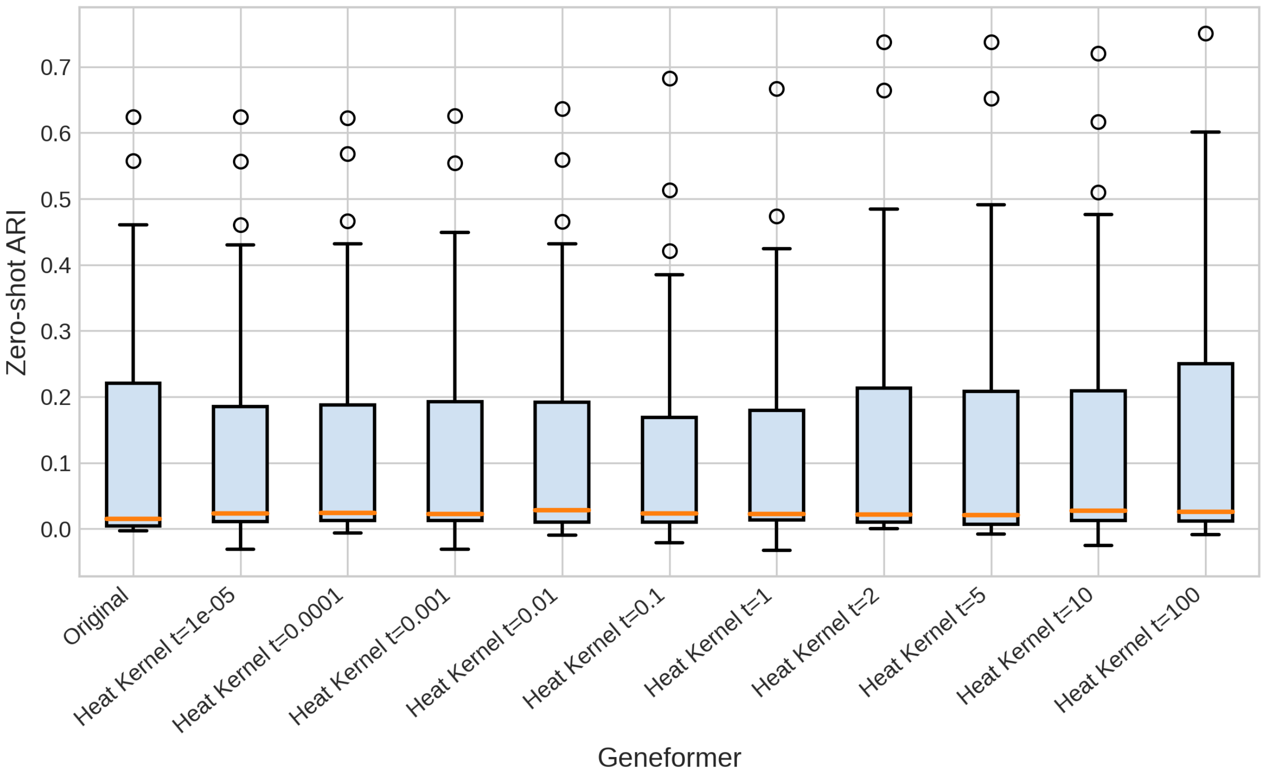

Figure S15: Zero-shot clustering ARI of different values of t for Geneformer over all datasets.

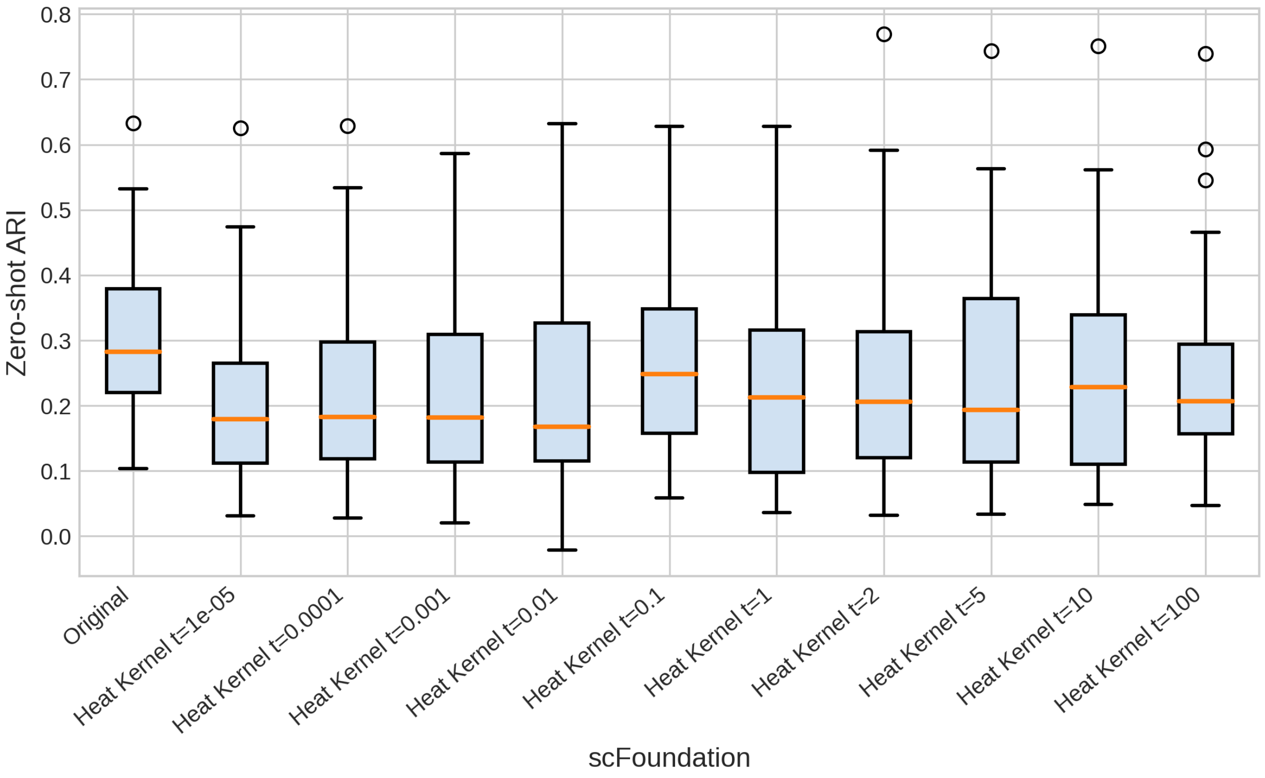

Figure S16: Zero-shot clustering ARI of different values of t for scFoundation over all datasets.

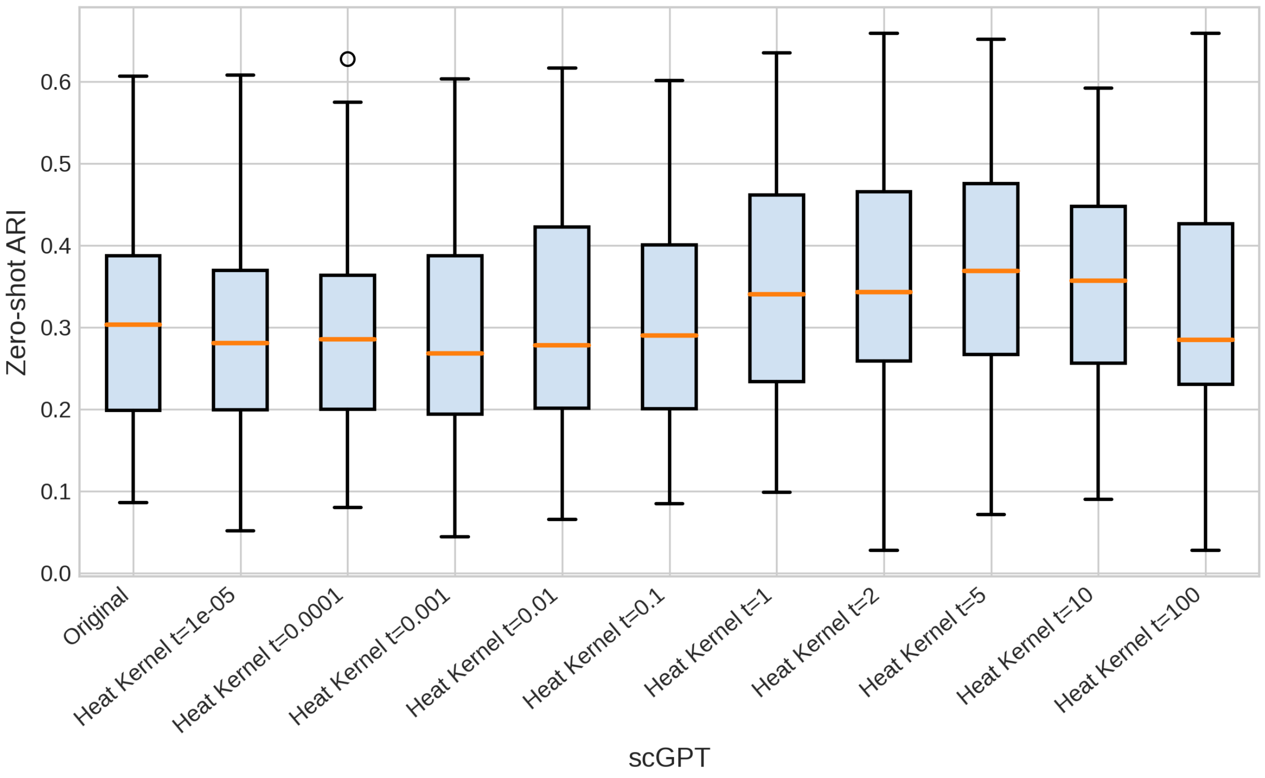

Figure S17: Zero-shot clustering ARI of different values of t for scGPT over all datasets.

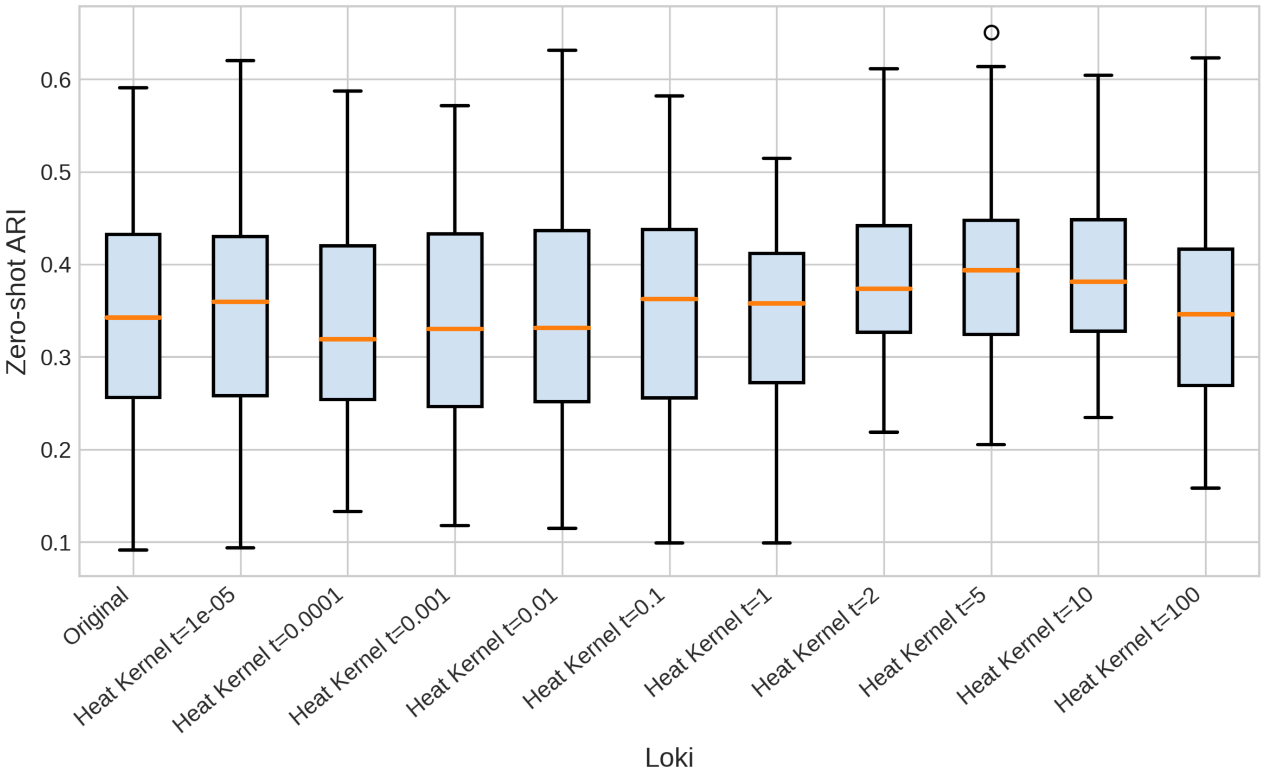

Figure S18: Zero-shot clustering ARI of different values of t for Loki over all datasets.

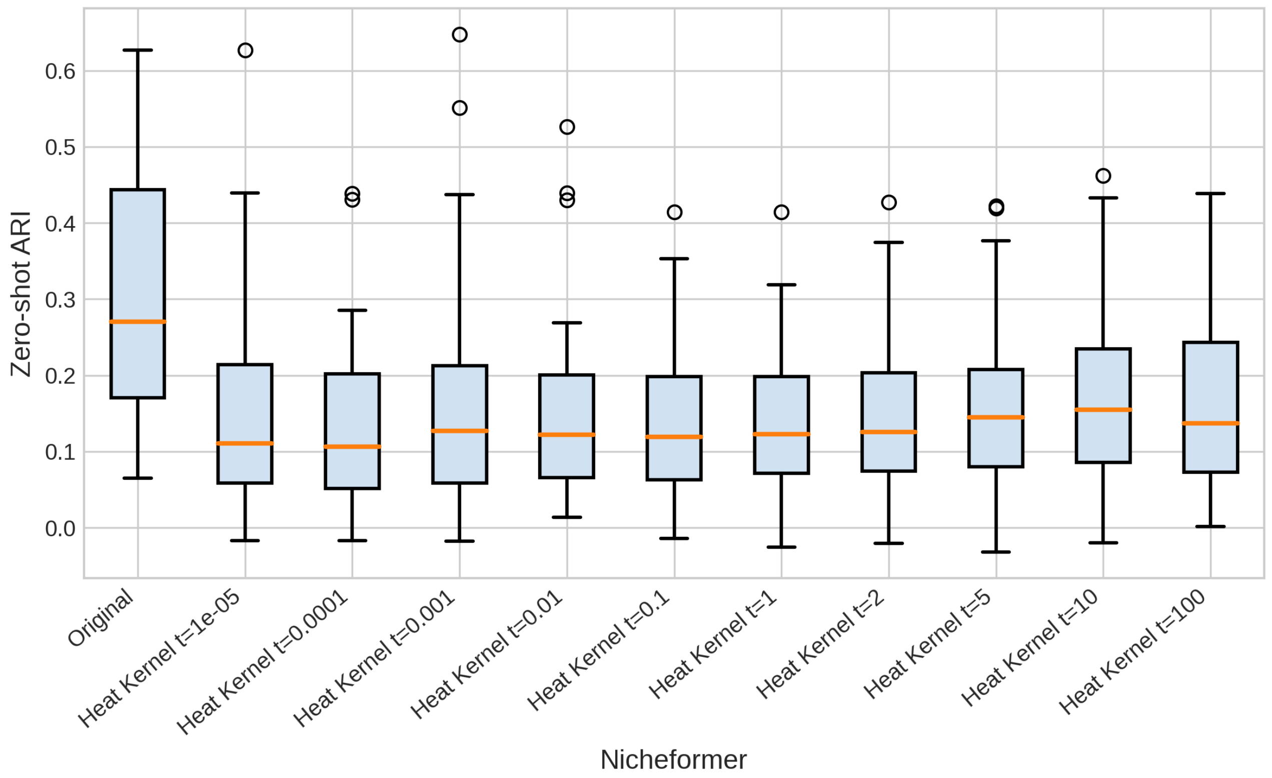

Figure S19: Zero-shot clustering ARI of different values of t for Nicheformer over all datasets.

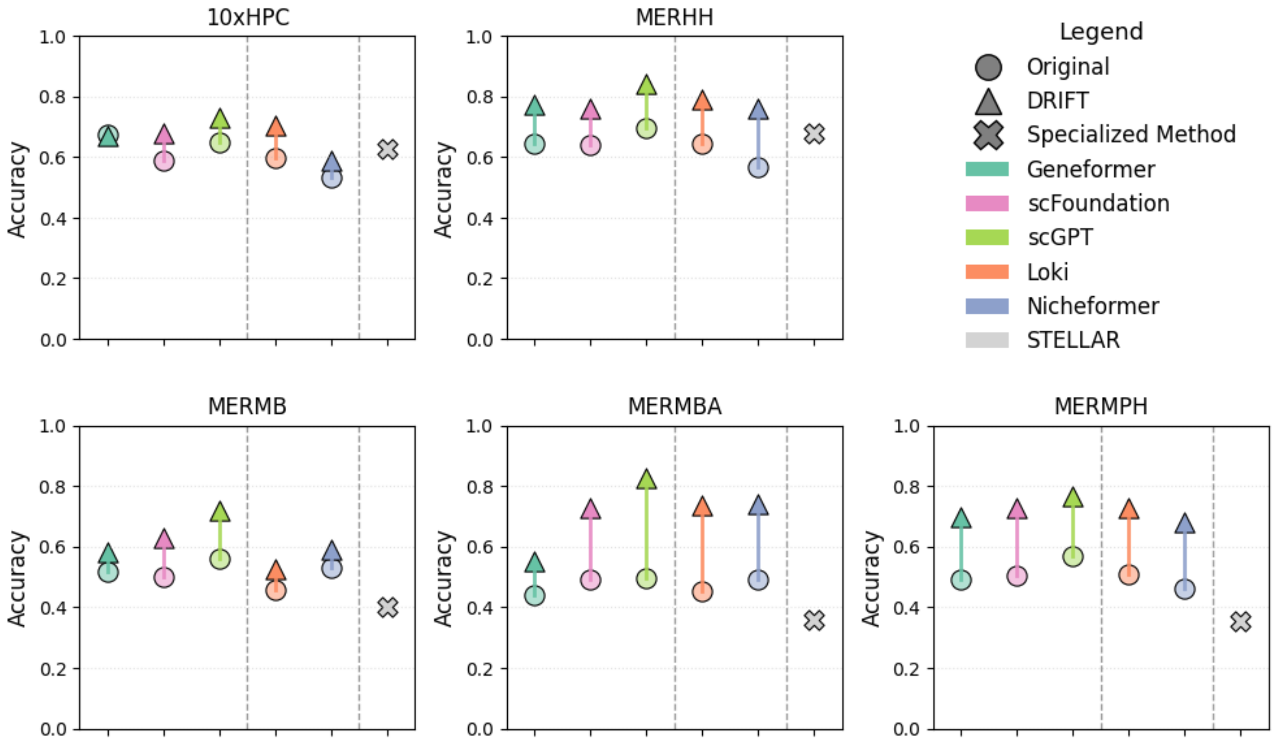

Figure S20: **Annotation Results** - A quantitative comparison across multiple datasets. The circles represent the performance of the original foundation models, the triangles represent the DRIFT-incorporated foundation models, and the X represents the specialized method STELLAR. The colors specify which column corresponds to which foundation model (the order of models in the legend is consistent with column placement). This figure demonstrates that DRIFT improves the F1-score performance of foundation models on the annotation task. Furthermore, DRIFT-incorporated foundation models, specifically scGPT show state-of-the-art performance, surpassing even the specialized model - STELLAR.

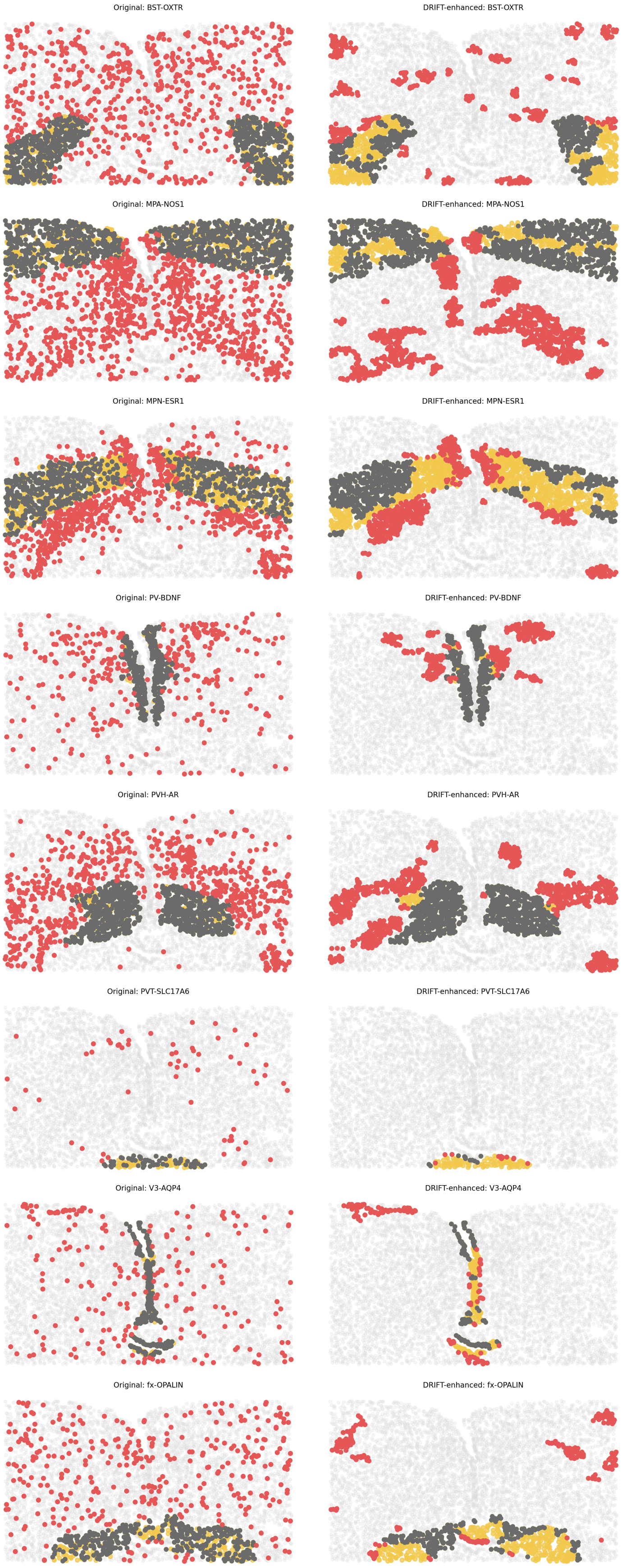

Figure S21: Effect of diffusion (DRIFT) on boundaries and marker genes.

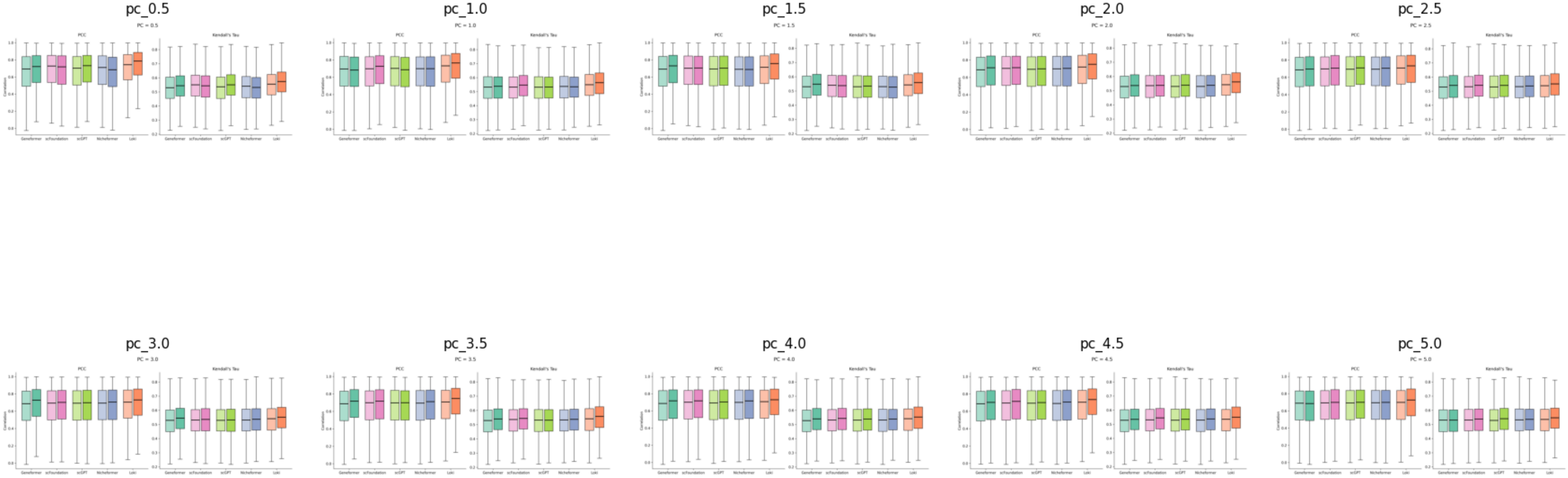

Figure S22: Effect of diffusion (DRIFT) on alignment performance across different pseudocount settings.

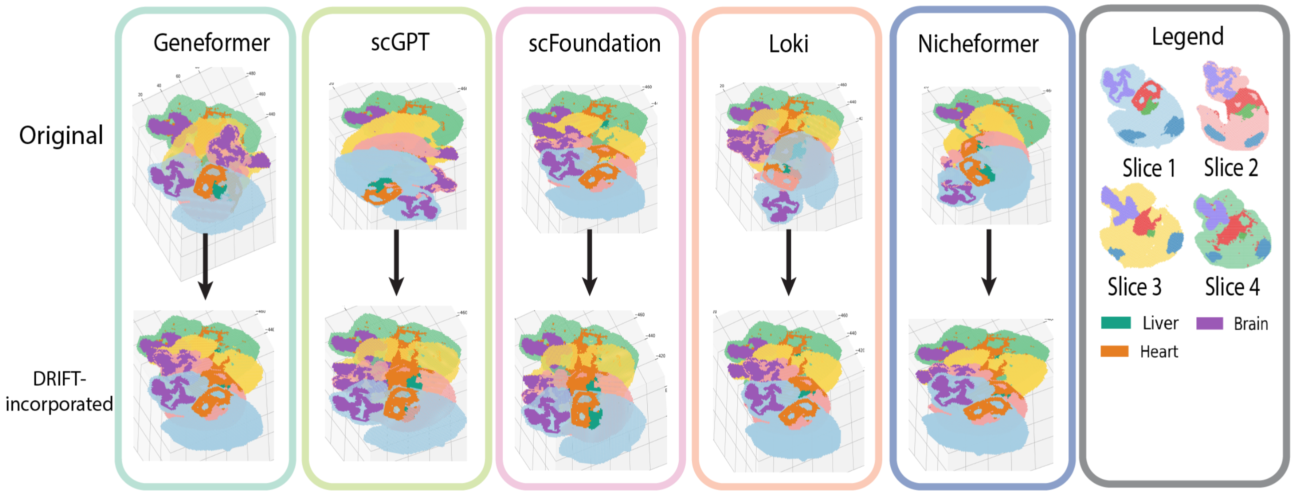

Figure S23: 3D reconstruction from all foundation models with and without DRIFT

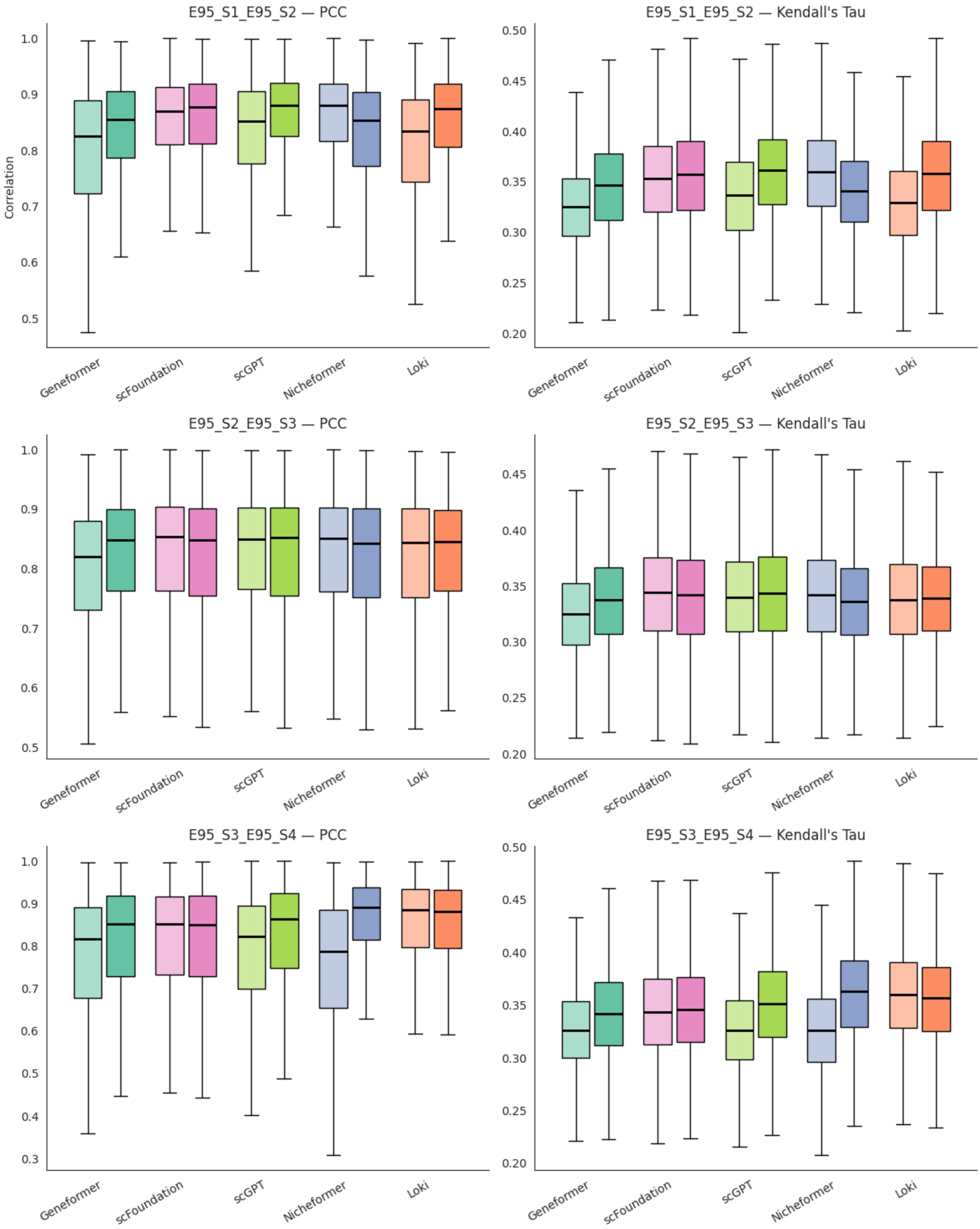

Figure S24: Effect of diffusion (DRIFT) on reconstructing 3D embryos.

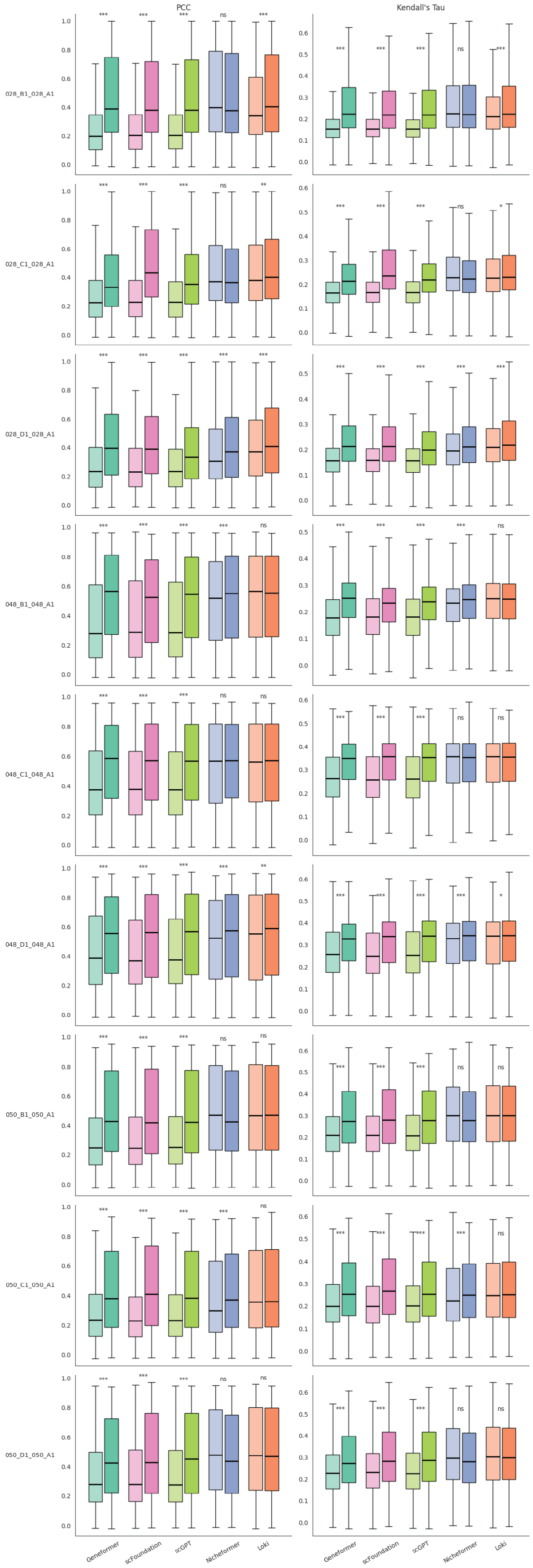

Figure S25: Full results of human intestine alignments.
